# Captivating nano sensors for mercury detection: a promising approach for monitoring of toxic mercury in environmental samples

**DOI:** 10.1039/d4ra02787f

**Published:** 2024-06-12

**Authors:** Nikkey, Suman Swami, Neelam Sharma, Ajay Saini

**Affiliations:** a Department of Chemistry, Chandigarh University NH-05, Ludhiana – Chandigarh State Hwy Mohali Punjab 140413 India sumanswami1994@gmail.com; b Department of Chemistry, Manipal University Jaipur Jaipur-Ajmer Express Highway, Dehmi Kalan, Near GVK Toll Plaza Jaipur Rajasthan 303007 India; c Central Analytical Facilities, Manipal University Jaipur Jaipur-Ajmer Express Highway, Dehmi Kalan, Near GVK Toll Plaza Jaipur Rajasthan 303007 India

## Abstract

Mercury, a widespread highly toxic environmental pollutant, poses significant risks to both human health and ecosystems. It commonly infiltrates the food chain, particularly through fish, and water resources *via* multiple pathways, leading to adverse impacts on human health and the environment. To monitor and keep track of mercury ion levels various methods traditionally have been employed. However, conventional detection techniques are often hindered by limitations. In response to challenges, nano-sensors, capitalizing on the distinctive properties of nanomaterials, emerge as a promising solution. This comprehensive review provides insight into the extensive spectrum of nano-sensor development for mercury detection. It encompasses various types of nanomaterials such as silver, gold, silica, magnetic, quantum dot, carbon dot, and electrochemical variants, elucidating their sensing mechanisms and fabrication. The aim of this review is to offer an in-depth exploration to researchers, technologists, and the scientific community, and understanding of the evolving landscape in nano-sensor development for mercury sensing. Ultimately, this review aims to encourage innovation in the pursuit of efficient and reliable solutions for mercury detection, thereby contributing to advancements in environmental protection and public health.

## Abstract 2

This review comprehensively provides insight into the extensive spectrum of nano-sensor development for mercury detection.

## Graphical abstract

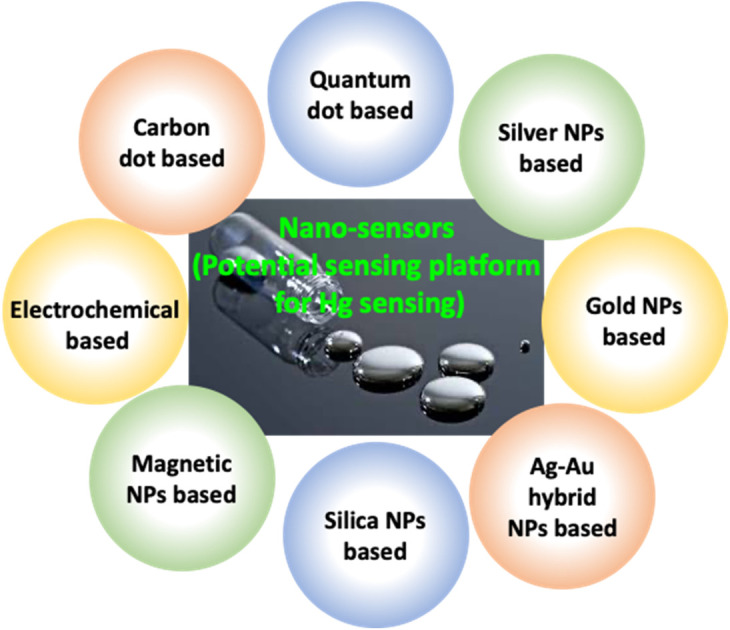

## 1. Introduction

Mercury contamination poses a substantial challenge to worldwide environmental preservation and public health.^1^ As per the US EPA 2015 report, the annual global emissions of mercury caused by human activity are estimated to reach 2220 metric tons, making mercury one of the most hazardous elements. It poses serious risks to ecosystems and human health.^2^ The soluble divalent mercury ion (Hg(ii)) is the most common and persistent type of mercury pollution in water, persisting in the environment because it doesn't break down naturally. Due to its non-biodegradable nature, once Hg(ii) is released into the environment, it cannot be eliminated.^3^ The subsequent accumulation of Hg(ii) in humans through the food chain can lead to damage to the lungs, brain, nervous system, gastrointestinal tract, kidneys, and endocrine system.^2,4,5^ Mercury ions experience a sequence of biogeochemical changes, leading to the formation of harmful chemical species. The toxicity of Hg(ii) primarily stems from its strong ability to bind with thiol (–SH) and amino (–NH_2_) groups present in proteins.^6,7^ This binding potential may lead to detrimental impacts on various bodily functions, including the pulmonary and kidney functions, central nervous system, chromosomes, and the immune system.^8,9^ Following the toxicity of mercury, the Minamata Convention on Mercury launched a reduction or phase-out of the use of mercury in industrial processes in 2017.^10–12^ Though, Article 3 (Annex A) of the Minamata Convention permits the utilization of mercury for indispensable purposes, including products necessary for civil protection and military applications.^12–14^ Consequently, safe management of natural source *via* monitoring the presence of mercury ions or pollution distribution in natural source has been an active area of research. Traditionally researchers employed various methods such as atomic absorption spectrometry,^15^ voltammetry,^16^ inductively coupled plasma mass spectrometry,^17,18^ inductively coupled plasma atomic emission spectrometry,^19^ spectrofluorometric,^20^ and chromatography.^21^ However, traditional analytical techniques for heavy metal detection are hindered by limitations such as complex protocols, expensive instrumentation, and the need for skilled personnel. Moreover, the detection of mercury, particularly at low levels in aqueous environments, remains a persistent challenge due to limitations inherent in conventional techniques. Conversely, simple, cost-effective, and time-efficient methods are also being utilized for metal detection, such as colorimetric and fluorescence detection *via* engaging scaffolds such as rhodamine-B,^22–26^ 1,8-naphthalimide,^27–29^ coumarin,^30–32^ pyrene,^33–35^ calixarene,^36,37^ and hydroxyquinolines^38–40^*etc.* Nevertheless, certain sensors exhibit limitations due to challenges in synthesis, expensive starting materials, or insufficient selectivity, and typically operate within organic solvents, thereby restricting their applicability in real environmental conditions. Consequently, in recent years, nanomaterial-based sensors have emerged as promising alternatives, capitalizing on their high surface reactivity, large surface area, size-dependent properties, high degree of functionalization and enhanced adsorption capacity.^41^ Nanomaterials, with their nanoscale dimensions (1–100 nm), exhibit unique properties such as high surface area, improved mechanical strength, and enhanced chemical reactivity, which traditional materials lack. These properties enable significant advancements in fields like energy storage, drug delivery, and environmental remediation. Nanostructured materials intensified sensitivity and selectivity, facilitating the precise easy detection of toxic mercury ions in environmental samples *via* colorimetric and optical changes. These advanced sensors hold the potential for rapid, sensitive, and cost-effective detection of aqueous Hg(ii) concentrations. Considering these developments, number of publications received in past as review articles to address the development such an group of researcher reviewed Metal–Organic Frameworks (MOFs) for mercury detection and removal,^42^ similarly, Xuyan Yan *et al.* reviewed MOFs for mercury detection,^43^ Sherif A. El-Safty *et al.* reviewed an short tutorial view on optical sensor for mercury,^44^ Dihua Dai *et al.* discussed some functional material for mercury detection and removal.^45^ Recently, a group reviewed polymeric chemosensors for mercury detection, but no one has discussed the wide range of nano sensor in detail at a single platform. So, in this literature review aims to comprehensively explore the latest utilization of various nanomaterials as sensors for mercury detection. Specifically, emphasis is placed on silver, gold, silica, magnetic, quantum dot, carbon dot, and electrochemical nanomaterials. By synthesizing existing research, this review seeks to provide in-depth exploration to researchers, technologists, and the scientific community, and understanding of the evolving landscape in nano-sensor development for mercury sensing.

## 2. Nano-sensors as potential detection approach for mercury ions

**Fig. 1:**
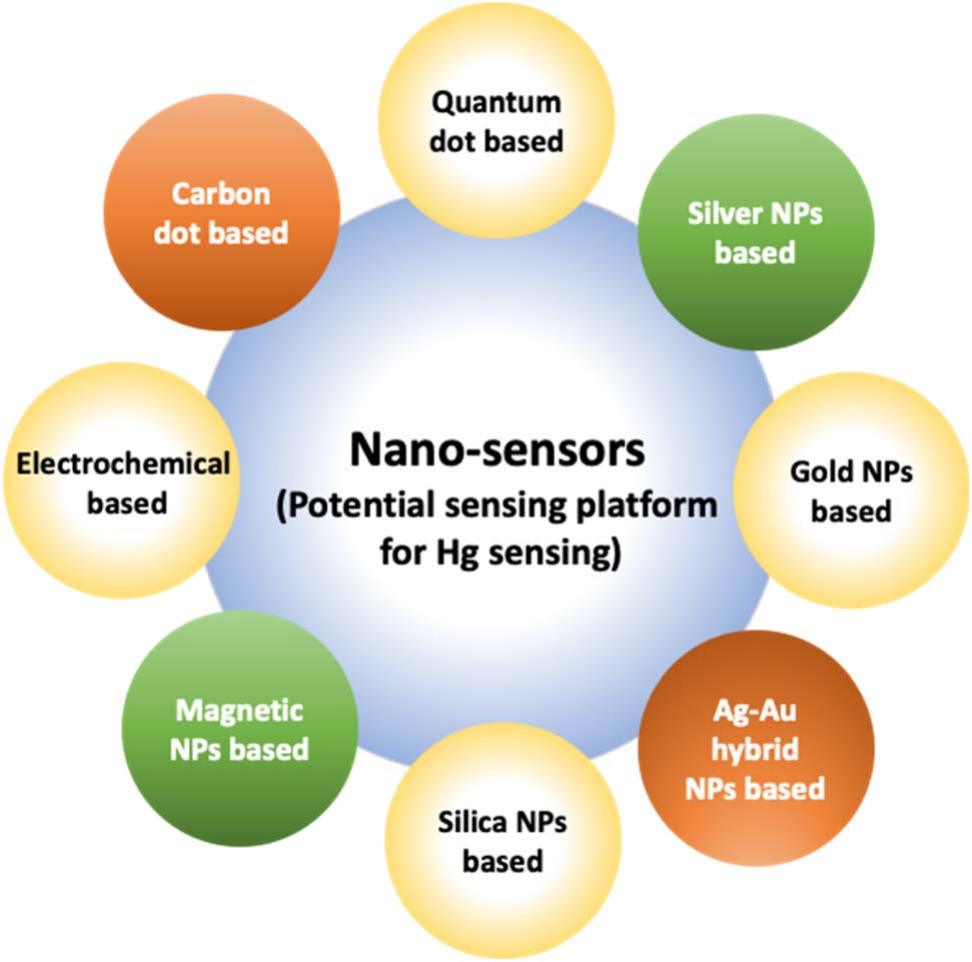
Various nano sensors serve as potential sensing platform for mercury sensing.

**Fig. 2:**
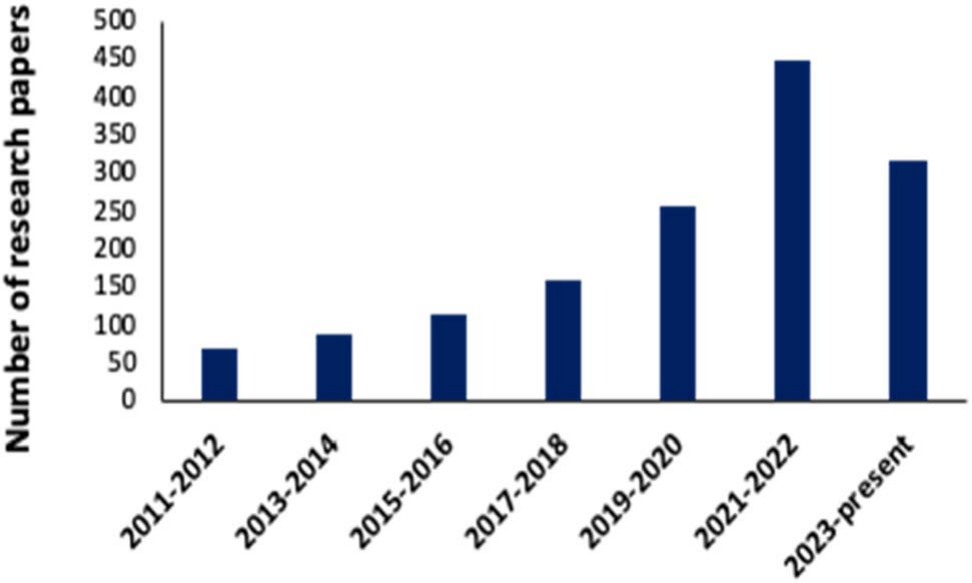
Progress in research papers: nanosensor for mercury detection (data collected from Science Direct, Google Scholar, Research Gate, and PubMed Central *etc.*) using key words: nanosensor for mercury sensing, Ag, Au, Ag–Au hybrid, magnetic silica-based, quantum dot, carbon dot and organic nanosensor for mercury detection.

A noticeable trend is observed from traditional detection methodologies towards nanosensors for identifying various hazardous substances. This shift is driven by the remarkable properties exhibited by nanomaterials in contrast to bulk materials. Particularly for detecting toxic heavy metal ions like mercury, there's a significant demand for precise sensing tools because even at low quantities, they are harmful.^46^ Nanosensors designed for mercury detection employ intricate mechanisms connecting the unique characteristics of nanomaterials to detect mercury at exceptionally low concentrations, often requiring minimal setup with advantages of specific and selective response based on the aggregation and morphology transition and SERS detection.^47–49^ Surface functionalization significantly enhances the affinity and selectivity of these nanomaterials towards mercury ions. A wide range of nanomaterials, including silver nanoparticles (AgNPs), gold nanoparticles (AuNPs), hybrid combinations like Ag–Au, silica nanoparticles (SiO_2_NPs), magnetic nanoparticles (MNPs), electrochemical materials, carbon-based nanostructures, and quantum dots, have been developed by researchers for this purpose (Fig. 1). The scientific community has witnessed an emergence in publications focusing on the development of mercury detection methods in recent years, indicating considerable potential in this field (Fig. 2).

### 2.1. Silver NPs based nano-sensors for mercury detection

Silver nanoparticles (AgNPs) are highly appealed after in sensing applications because they leverage distinct absorbance band at certain resonant frequencies characterized in the form of localized surface plasmon resonance (LSPR), allowing their use for selective and sensitive response towards analytes. LSPR is an optical phenomenon generated by a light wave trapped within conductive nanoparticles (NPs) smaller than the wavelength of light.^50^ By capitalizing on the excellent conductivity, high surface area, and versatile chemistry of silver-based nanomaterials, these sensors demonstrate promising capabilities in colorimetric recognizing and quantifying mercury ions either *via* aggregation or optical changes based on etching processes. Functionalization strategies, including surface modifications and the integration of specific ligands, enhance the affinity of AgNPs for mercury, thereby improving selectivity. The optical properties of AgNPs also enable diverse sensing mechanisms, facilitating efficient and often rapid detection of mercury in various environments. For instance, a nano sensor based on silver nanoparticles (AgNPs) was developed by Aminu Oladepo *et al.* for mercury sensing using an eco-friendly synthetic method. The AgNPs were synthesized rapidly in just in 60 seconds using orange peel extract and thoroughly characterized using UV-visible, FESEM, FTIR, and XRD techniques. The synthesized AgNPs exhibited a “surface plasmon resonance” (SPR) absorption band at 420 nm and colors ranging from yellowish brown to golden brown. FESEM images depicted polydisperse irregularly shaped AgNPs with an approximately average size of 55 nm. XRD analysis confirmed the presence of characteristic peaks of silver, whereas FTIR spectra identified functional groups responsible for reducing silver ions and stabilizing the NPs. The AgNPs colloid solution functioned as a nanosensor for visually detecting Hg(ii) ions in water. Successively, upon adding Hg(ii) ions led to the color change of the golden brown AgNPs colloid solution to colorless, accompanied by the disappearance of the characteristic SPR absorption band. The AgNPs revealed notable selectivity and sensitivity for colorimetric detection of Hg(ii) ions, and demonstrating a detection limit 0.25 ppm (1.24 × 10^−6^ mol L^−1^), with a linear response at concentrations ranging from 1–100 μM.^51^

**Fig. 3:**
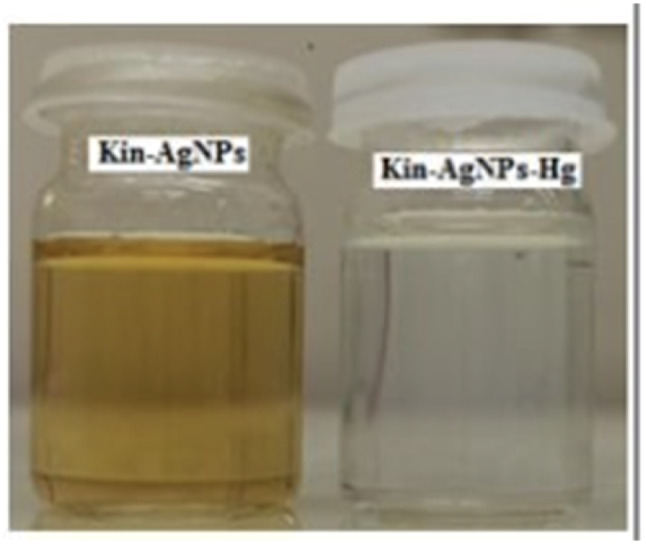
Colorimetric changes in Kin-AgNPs after addition of Hg(ii) ions (yellow to colorless) (reproduced from ref. 52 with permission from Royal Society of Chemistry, Copyright @ 2021).

**Scheme 1:**
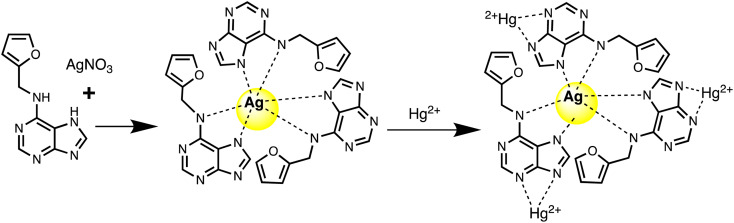
Graphic for Kin-AgNPs synthesis and Hg(ii) detection.

Similarly, a kinetin-based nanoparticles (Kin-AgNPs) were developed *via* a chemical reduction method and comprehensively analyzed using spectroscopic techniques to function as a Hg(ii) sensor. These Kin-AgNPs exhibited exceptional stability under various conditions, including high electrolyte concentrations, higher temperatures, and a broad pH range. Upon adding mercury, the absorbance intensity of Kin-AgNPs decreased due to quenching with colorimetric changes (Fig. 3), which indicating the binding of Hg(ii) ions with the nitrogen or oxygen atoms of NPs through their lone pairs. Notably, Kin-AgNPs displayed high selectivity, even amidst the presence of multiple competing metal ions. Absorption intensity showed a straight linear association with Hg(ii) concentration from 0.01 to 100 μM. The developed nano sensor (Kin-AgNPs) exhibited a detection limit (LOD) of 6.6 nM for Hg(ii) ions. Additionally, Kin-AgNPs were effectively utilized for detecting mercury in laboratory tap water where the chemo sensors are crucial to detect the analyte (Scheme 1).^52^

**Fig. 4:**
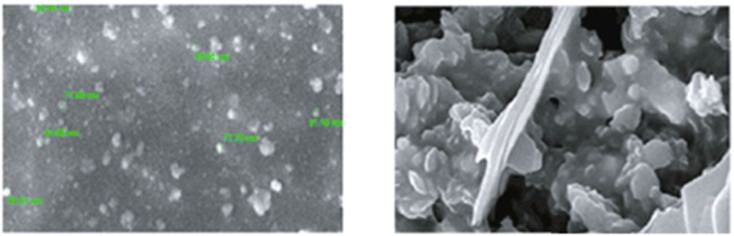
SEM images of ACR-AgNPs (as prepared and after addition of Hg(ii) ions) (reproduced from ref. 54 with permission from Hindawi, Copyright © 2022).

Further in a report, Balasurya *et al.* devised PVP-stabilized AgNPs-methionine for detecting Hg(ii) ions in aqueous samples at the nanomolar level. The inclusion of methionine in the AgNPs-methionine complex was crucial for mercury sensing due to the greater affinity of Hg(ii) ions towards the sulfur in methionine. AgNPs-methionine aggregated upon the addition of Hg(ii) ions, leading to a noticeable color shift from pale-yellow to colorless. Analysis of particle size validated the modification in the nanoparticle structure upon interaction with Hg(ii) ions. Additionally, detection of Hg(ii) was achieved through paper strip and agarose gel methods.^53^ In a parallel approach, silver nanoparticles functionalized with acridine (ACR-AgNPs) were utilized as a nanosensor to accurately detect and quantify Hg(ii) ions present in tap water. The assessment of the interaction between the Hg(ii) ions and NPs was conducted through UV-visible and FT-IR spectroscopy. Additionally, morphological characteristics and particle size were analyzed using AFM, DLS, and SEM techniques. Upon the addition of Hg(ii) to the ACR-AgNPs solution, a decrease in absorbance intensity was observed, indicating the binding of Hg(ii) ions with the nitrogen atoms of ACR-AgNPs *via* their lone pairs and induce an agglomeration in the NPs as depicted in Fig. 4. Stable complexes were presumed to form between Hg(ii) ions and the Schiff base nitrogen atoms of ACR-AgNPs, disrupting the electronic environment and causing a significant decrease in absorption intensity. The ACR-AgNP-based nanosensor exhibit LOD of 1.65 μM across a wide pH range and concentration linear ranges of 5–100 μM. Notably, in the presence of other interfering ions, the proposed mercury sensor also demonstrated efficient performance.^54^

Haji *et al.* employed a green synthetic approach to synthesize AgNPs using tree gum as a dual reducing and stabilizing agent. The next step was using these nanoparticles as a colorimetric sensor to detect mercury ions. Almond gum coated AgNPs (AgNPs@AG) exhibited remarkable sensitivity and facilitated the colorimetric detection of Hg(ii) ions in water samples. The detection method relied on the aggregation of AgNPs, leading to the disappearance of their yellow color, which was monitored using a spectrophotometer. The observed LOD was 0.5 mg L^−1^. Consequently, in an aqueous medium, AgNPs@AG displayed rapid and high sensitivity to Hg(ii) ions.^55^ Likewise, biogenic AgNPs were synthesized using plant extract sourced from basil and analyzed *via* spectroscopic techniques. The synthesized spherical AgNPs exhibited notable selectivity in detecting Hg(ii) ions compared to other cations, displaying high sensitivity across different concentrations of Hg(ii). During colorimetric analysis, it was perceived that adding 1 mL of AgNPs to 9 mL of 1 mM Hg(ii) solution transformed the previously colorless Hg(ii) solution into a golden-brown hue. However, within 5 minutes, the color changed gradually from golden brown to light brown, then to colorless. This color change suggested that Hg(ii) ions had a considerable effect on the AgNPs' SPR vibration. The observed color change could be attributed to AgNP aggregation and subsequent decolorization owing to the formation of Ag–Hg amalgam. The detection limit for Hg(ii) was 12 μg L^−1^ (6.25 × 10^−8^ mol L^−1^), demonstrating the great sensitivity of these biogenically synthesized AgNPs as a tool for detecting Hg(ii) ions.^56^

**Scheme 2:**
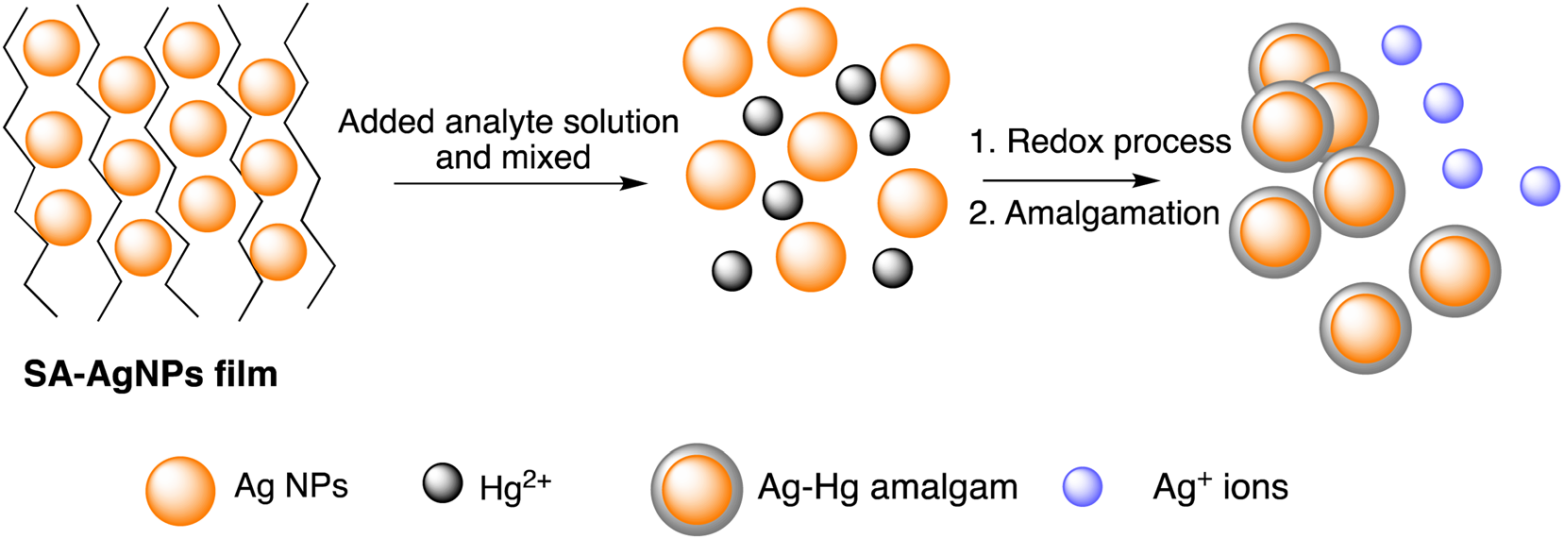
Schematic representation of Hg sensing *via* SA-AgNPs film.

Further, Imran Uddin *et al.* assessed a biosynthetic approach for AgNPs development utilizing *Matricaria recutita* plant's extract. The resulting NPs were characterized using XRD, UV-visible, TEM, and FTIR analysis before being employed for visual colorimetric detection of Hg(ii) ions. TEM examination revealed that the synthesized AgNPs with a typical size of the particles of 11 nm exhibit quasi-spherical appearance. The absorbance intensity of AgNPs reduced once interacting with Hg(ii) ions, causing a color shift at a concentration of 10 ppm from yellowish-brown to colorless. Furthermore, in the UV-visible spectra, the signal for AgNPs was not seen when mercury ions interacted with the nanoparticles.^57^ The solvent casting method was used to synthesize a composite nanosensor composed of SA–alginate–AgNPs. This nanosensor was employed to detect trace Hg(ii) ions on a colorimetric basis and *via* naked eyes. Structural properties of the fabricated nano sensor were determined using instrumental methods. The results indicated that AgNPs were formed on average with a diameter of 13.34 nm. The colorimetric sensing Hg(ii) of the nanosensor was performed under specific conditions and showed a linear correlation of the absorbance (402 nm) of the nanosenor to the Hg(iii) ion concentration (0.025–60 μM). The produced composite nano sensor made up of AgNPs was used to detect Hg(iii) ions as shown in Scheme 2. The LOD of the synthesized nanosensor was 5.29 nM. Furthermore, this sensor effectively detected Hg(ii) ions with recoveries ranging from 81.58% to 114.73% in environmental samples.^58^

**Fig. 5:**
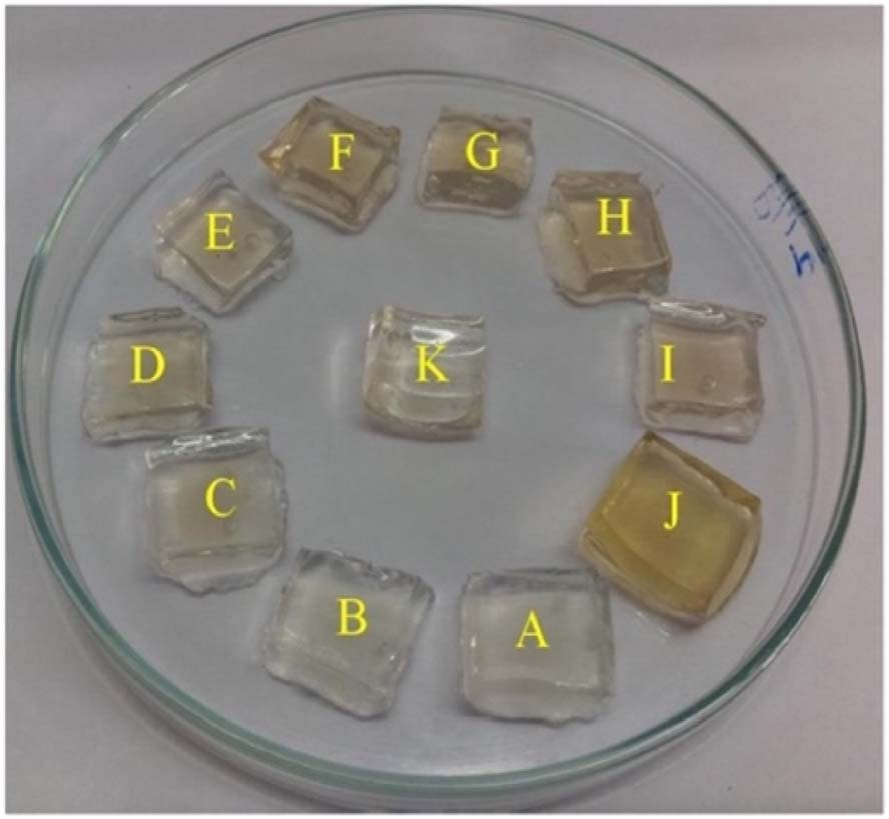
Color changes of hydrogel with Hg(ii) ions (0–20 minutes) (reproduced from ref. 61 with permission from Elsevier Ltd, Copyright © 2016).

Additionally, a SERS nanosensor based on 4-mercaptopyridine (4-MPY) functionalized AgNPs (4-MPY-AgNPs) has been developed to detect Hg(ii) ions in the presence of spermine. Spermine binds AgNPs through Ag–N bonds, resulting in significant AgNP aggregation and significantly increased Raman intensity for the reporter molecule. Upon addition of Hg(ii), Hg–Ag formation blocks 4-Mpy and spermine adsorption, resulting in 4-Mpy-Ag dispersion and decreased Raman intensity for SERS detection. A fine linearity was noticed in the range 1–100 nM with high detectability (LOD = 0.34 nM) of SERS responses due to spermine induced AgNPs aggregation.^59^ A photo induced green crystalline AgNPs has been developed using the bioligands contained in the extract of *Allium sativum* (garlic) (bioligands) act as stabilizing as well as reducing agents. The presence of light sources in the environment improves the process of nanoparticle formation. The synthesized NPs demonstrate excellent sensitivity with LOD of 2 μM for Hg(ii) ions. An alteration in the LSPRs of silver nanoparticles occurs which was characterized by a blue shift when increasing concentration of Hg(ii) ions added into the AgNPs solution. This shift leads to a transition in the solution's color, shifting from yellow to a colorless hue. This change is due to the oxidation process (Ag^0^ → Ag^+^) and the reduction process (Hg^2+^ → Hg^0^).^60^ Similarly, Jeevika and Shankaran *et al.* developed gelatin functionalized AgNPs *via* a chemical method. The colorimetric detection of Hg(ii) was performed across three distinct phases: “solution, paper substrate and hydrogel network”. After the inclusion of Hg(ii) to the AgNPs solution, aggregation occurs, resulting color shift in color from yellow to colorless due to the establishment of Ag/Hg amalgam. AgNPs probe demonstrated notable sensitivity (LOD = 25 nM) for Hg(ii) ions under optimal conditions. 3D hydrogel matrix and disposable paper strips were also evaluated for sensing Hg(ii) ions. As depicted in Fig. 5, AgNPs/PVA hydrogels were exposed to varying concentrations of Hg(ii) solution, leading to a color change from yellow to colorless on the outer layer of the hydrogel within 20 minutes, attributed to a redox reaction (Ag/Hg amalgam formation). In both conditions, the sensor exhibited comparable sensitivity and specificity for Hg(ii) detection under optimized conditions, demonstrating consistent performance across the board. This gelatin-functionalized nano probe demonstrates high versatility and holds promise for mercury detection in real applications.^61^

**Fig. 6:**
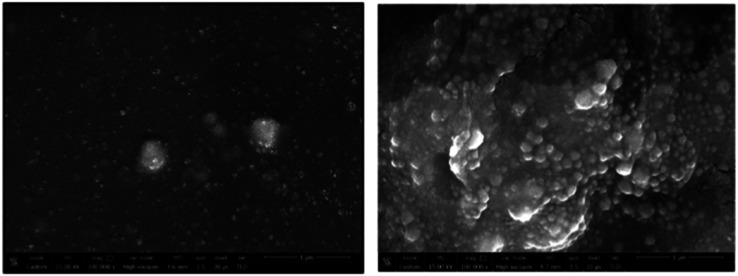
SEM images of Mod-Ch–AgNPs as prepared and after addition of Hg(ii) ions (reproduced from ref. 62 with permission from Elsevier Ltd, Copyright @ 2018).

In a study, Chitosan–capped AgNPs (Ch–AgNPs) have been developed for mercury sensing. The initially yellow-colored Ch–AgNPs immediately turned colorless within 10 seconds upon addition of Hg(ii) ions and exhibit an agglomeration in NPs as depicted in Fig. 6, SEM images of NPs after addition of Hg(ii) ions. Similarly, Cu(ii) and Fe(iii) ions also resulted in a colorless solution after 1 minute. To moderate interference from Cu(ii) and Fe(iii), the amine group of chitosan and the carboxylic acid group of 3-mercaptopropanoic acid reacted to produce thiol-terminated chitosan through an amide coupling process. Sensing analysis revealed that thiol-terminated Ch–AgNPs (Mod-Ch–AgNPs) are highly selective and rapid for detecting Hg(ii) ions. Colorimetric analysis demonstrated that upon addition of Hg(ii) ions to the Mod-Ch–AgNPs solution, the solution turned colorless within 5 seconds, with a LOD of 5 ppb.^62^ Immediate colorimetric response and low LOD makes promising to the Mod-Ch–AgNPs in the real samples for mercury sensing compared to other reported methods.

**Fig. 7:**
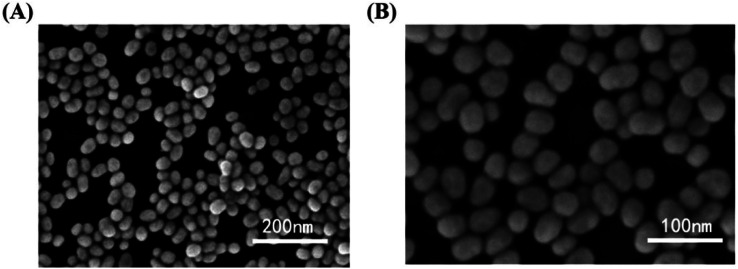
AgNPs SEM images in magnification of (A) 50 000× (B) 100 000× (reproduced from ref. 63 with permission from Elsevier B. V., copyright © 2020).

Similarly, a SERS sensor have developed by Zhao *et al.* for mercury sensing by decorating the inner wall of a capillary with 4,4′-dipyridyl (Dpy) functionalized AgNPs. The prepared greenish-yellow AgNPs exhibit spherical shape with average size of about 30 nm, as revealed in SEM images (Fig. 7). There is a uniform distribution of NPs. In the presence of Hg(ii), the SERS signal decreases because the Dpy molecules detach from the surface of AgNPs and coordinate with Hg(ii) ions. A LOD of 0.1 ppb was achieved, demonstrating a linear correlation between Raman intensity and Hg(ii) concentrations spanning from 1 to 100 ppb, facilitating accurate quantitative analysis. The sensor demonstrates good reproducibility and selectivity, successfully detecting Hg(ii) in real environmental water samples.^63^

**Fig. 8:**
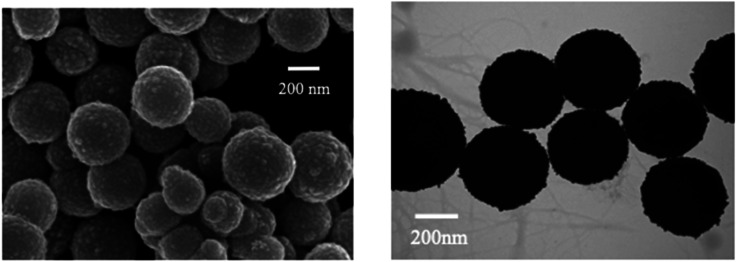
SEM and TEM mages of as prepared Fe_3_O_4_@AgNPS (reproduced from ref. 64, with permission from Royal Society of Chemistry, Copyright @ 2017).

Furthermore, Song *et al.* demonstrated a Fe_3_O_4_@Ag-based label-free SERRS nanosensor for the selective detection of Hg(ii) ions. The sensor's functionality relies on the competitive interaction between Hg(ii) ions and malachite green (MG) with nano-silver immobilized on Fe_3_O_4_@Ag magnetic beads (MBs) which are spherical in shape (Fig. 8). Furthermore, in the absence of Hg(ii), the Raman signal intensity of MG undergoes substantial enhancement because of its absorption onto the nano-silver surface *via* a single nitrogen atom. Conversely, when Hg(ii) is present, a redox reaction takes place between the zero-valent nano-silver and Hg(ii), resulting in the formation of an Ag/Hg amalgam on the surface of Fe_3_O_4_@Ag. Because of this interaction, MG's adsorption on the nano-silver surface is inhibited, which causes MG's SERRS signal intensity to decrease proportionately as Hg(ii) concentration rises. Upon optimization, the proposed label-free nanosensor exhibited unparalleled sensitivity in detecting Hg(ii) ions, reaching 10 pM (2 ppt), coupled with exceptional selectivity^64^

**Fig. 9:**
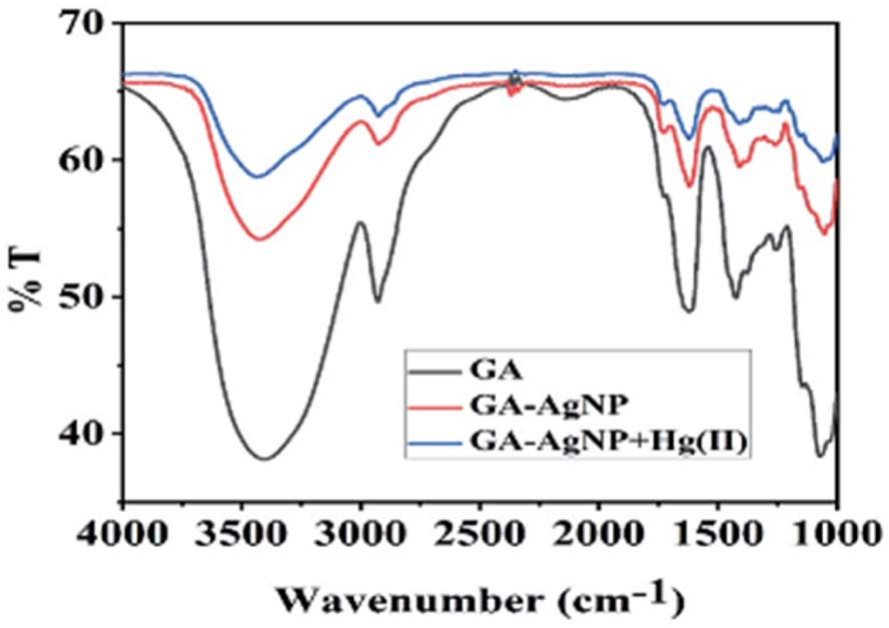
FTIR spectrum of gum acacia, gum acacia@Ag and gum acacia@Ag–Hg(ii) (reproduced from ref. 65 with permission from Royal Society of Chemistry, Copyright @ 2020).

Abbasi *et al.* have developed a dual sensor system utilizing gum acacia-mediated AgNPs to detect Hg(ii) through fluorescence “turn-on” and colorimetric responses, while also serving as a fluorescence “turn-off” sensor for malachite green. The researcher proposed a plausible mechanism elucidating the dual response mechanism towards Hg(ii) ions. FT-IR analysis suggests that Hg(ii) first interacts with gum acacia through its –COOH and –OH groups (Fig. 9). Gum acacia and mercury combine to generate a complex because of this interaction. An Ag@Hg nanoalloy is produced when the concentration rises because Hg(ii) ions quickly adsorb onto the surface of the NPs, causing a redox reaction between Ag(0) and Hg(ii) to generate Ag(i) and Hg(0) as well as an electron transfer from gum acacia to Ag(i). The intensified fluorescence signal is selectively quenched by the nanosensor due to the formation of Ag_2_S and HgS. This reported nanosensor demonstrates effectiveness in detecting malachite green *via* the inner filter effect. The linear detection ranges span from 3 nmol L^−1^ to 13 mmol L^−1^ for Hg(ii), with a corresponding LOD of 2.1 nmol L^−1^ for Hg(ii).^65^

In a colloidal solution of silver nanoparticles (AgNPs), shining a portable laser pointer pen (635 nm) creates a bright red Tyndall effect (TE). Huang *et al.* utilized this scattering signal of AgNPs' TE for highly sensitive visual detection of Hg(ii) ions at the point of need. With the addition of Hg(ii), the silver nanoparticles (AgNPs) degrade because of certain redox interactions between the analyte ions and the nanoprobes. This leads to a notable reduction or even disappearance of the Tyndall effect (TE) in the final reaction mixture. By visually assessing the TE intensity, qualitative analysis of Hg(ii) can be conducted, reaching down to about 5 nM. With a smartphone, precise quantitative readout for mobile imaging measurement can be further attained. The outcomes show that the TE-inspired assay (TEA) without any equipment can detect Hg(ii) linearly in a concentration range of 5 nM to 4 μM. With AgNP probes, the analyte's detection limit was estimated to be as low as 0.85 nM, providing a ∼5400-fold increase in assay sensitivity compared to conventional SPR-based colorimetric nanosensors.^66^ Recently, Chinmayee Pattnaik *et al.* have described the use of tulsi leaf extract and glucose-capped AgNPs for the detection of Hg(ii) ions in water. The NPs' LOD for Hg(ii) ions was 2.8 ppb with the optical modifications, which is better than the LOD of several other sensors based on green AgNPs that have been available in the literature.^67^ Additionally the AgNPs derived sensors more effective in term of lower limit of detection, long term reliability, low interference, without the need of complicated set-up and working in real samples, that was crucial with the many reported sensors in the past, such as for an comparative analysis some previously reported chemo sensor listed in Table 1, *via* comparison of Tables 1 and 2 (a comparative overview of Ag based nanosensor) it has clearly concluded that silver derived sensors exhibits better results than the other chemo sensors.

**Table 1:** Chemo sensors for mercury sensing

S. No.	Chemo sensor	LOD	Sample medium	Ref.
1	(*E*)-1-((5-(4-Nitrophenyl)furan-2-yl)methylene) semicarbazone	2.084 × 10^−9^ M	DMSO/H_2_O (8 : 2, v/v)	Q. Lin *et al.*^68^
2	1*H*-imidazo[4,5-*b*]phenazine derivatives	1.6 × 10^−7^ M	DMSO/H_2_O (6 : 4 /v/v)	J. Liu *et al.*^69^
3	2,6-Bis(aminoethyl)pyridine derived	1.0 × 10^−8^ M	DMSO and water (1 : 1, v/v)	L. Feng and Z. Chen^70^
4	Thiocarbohydrazide derived chemo sensor	1.26 nM	Semi-aqueous medium	R. Bhaskar and S. Sarveswari^71^
5	5-(2-Benzothiazolyl)-2-hydroxybenzaldehyde	14.3 nM	CH_3_CN : HEPES (70/30, v/v)	D. Aydin and I. Yilmaz^72^
6	Coumarin derived chemo sensor	1.91 × 10^−7^ M	DMF : H_2_O (2 : 8, v/v)	M. Gosi, A. C. Kumar and Y. Sunandamma^73^
7	Coumarin-thiourea conjugate	1.46 × 10^−7^ M	2 : 8 EtOH/H_2_O	X Zhang *et al.*^74^
8	Coumarin-thiol-based sensor	5.01 × 10^−8^ M	Aqueous	Shaily, A. Kumar and N. Ahmed^75^

**Table 2:** An overview of silver-based nano-sensors for sensing of mercury metal ion

Entry	Nano sensor	Sensing approach	Linear range	LOD	Ref.
1	AgNPs colloid	Colorimetric	1–100 μM	0.25 ppm	51
2	Kin-AgNPs	Fluorescence quenching	0.01 to 100 μM	6.6 nM	52
3	ACR-AgNPs	SERS	5–100 μM	1.65 μM	54
4	AgNPs@AG	Colorimetric	—	0.5 mg L^−1^	55
5	Biogenic AgNPs	Colorimetric	—	12 μg L^−1^	56
6	Sodium alginate-AgNPs	Colorimetric	0.025–60 μM	5.29 nM	58
7	4-Mercaptopyridine functionalized AgNPs	SERS	1–100 nM	0.34 nM	59
8	Green crystalline AgNPs	Colorimetric and LSPR	—	2 μM	60
9	Gelatin functionalized AgNPs	Colorimetric	—	25 nM	61
10	Chitosan capped AgNPs	Colorimetric	—	5 ppb	62
11	4,4′-Dipyridyl (Dpy) functionalized AgNPs	SERS signal	1 to 100	0.1 ppb	63
12	Fe_3_O_4_@Ag	SERRS signal	—	10 pM	64
13	Gum acacia-mediated AgNPs	Fluorescence turn-on and colorimetric	3 nmol L^−1^ to 13 mmol L^−1^	2.1 nmol L^−1^	65
13	AgNPs' TE	TE signal	5 nM to 4 μM	∼0.85 nM	66
14	Glucose-capped AgNPs	Optical changes	10–100 ppb	2.8 ppb	67

### 2.2. Gold NPs based nano-sensors for mercury sensing

**Fig. 10:**
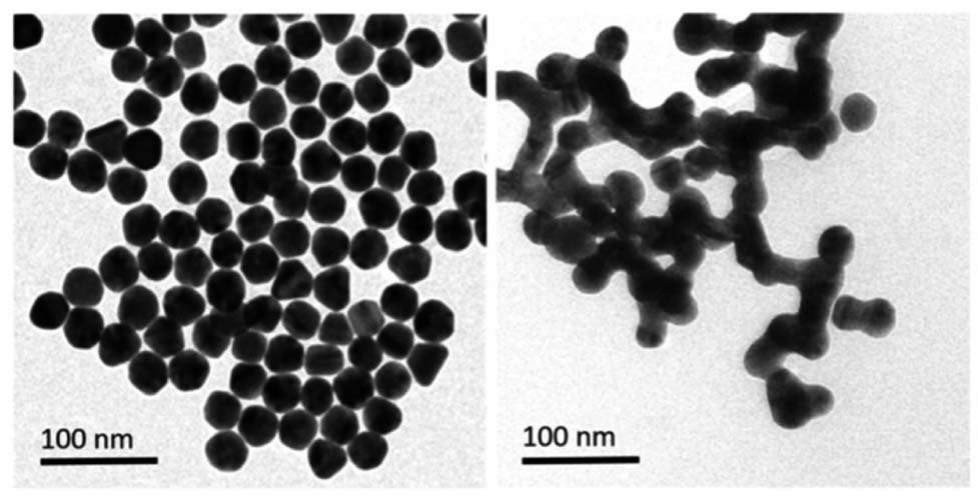
Illustrative TEM pictures of gold nanoparticles pre- and post-incubation with a sample calculatedly contaminated with methylmercury (reproduced from ref. 76 with permission from Wiley-VCH Verlag GmbH & Co. KGaA, Weinheim, Copyright © 2019).

Gold's excellent biocompatibility, stability, and facile functionalization make it an ideal candidate for designing nano sensors with enhanced performance. The distinctive surface plasmon resonance of gold nanoparticles can be exploited for colorimetric detection, enabling a visual response to the presence of mercury ions. Additionally, the high surface-to-volume ratio of gold nanostructures provides many active sites for binding with mercury ions, contributing to high sensitivity. For example, gold nanoparticles (AuNPs) effectively catalyze the conversion of organic mercury to its metallic form (Hg^0^), promoting nucleation and the formation of an amalgam on the surface of the particles. This process leads to a shift in plasmon resonance induced by aggregation. This procedure allows for quick and precise colorimetric identification of mercury species within a duration of 60 seconds. The LOD was achieved 20 ppb. TEM imaging (as depicted in Fig. 10) showcases the AuNPs both prior to and following the detection reaction with methylmercury. Initially, on the grid, the AuNPs are evenly distributed and distinct, but after methylmercury incubation, the formation of mercury amalgam becomes apparent, evidenced by the presence of large particle aggregates stuck together. This implies a possible alteration in color of the AuNPs suspension caused by an aggregation-triggered shift in plasmon resonance. A redox reaction between formic acid and methylmercury that takes place at the surface of Au nanoparticles starts the detecting process. When reduced elemental mercury (Hg^0^) forms on the surface of AuNPs, the particles quickly aggregate and the solution's color changes from red to violet.^76^

**Scheme 3:**
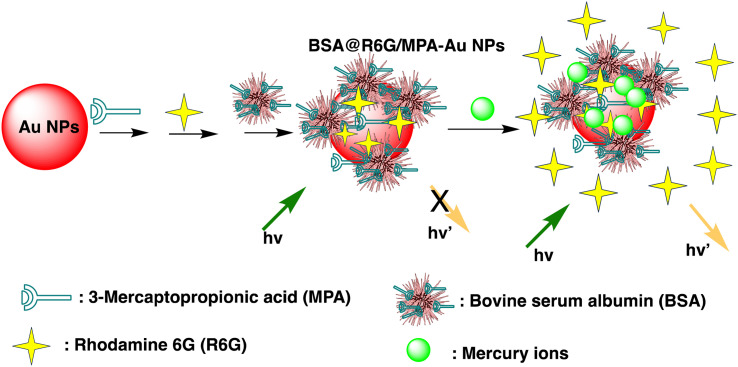
Schematic of BSA@R6G/MPA-Au NP preparation and Hg(ii) ions sensing.

Huizhen Yuan *et al.* formulated gold nanoparticles (AuNPs/T) modified with thymine (T) to serve as amplification tags for mercury detection. Upon optimization, it was noted that the change in resonance wavelength of SPR intensified with higher concentrations of Hg(ii) ions. In the detection process, Hg(ii) ions function as a bridging link. Initially, they are captured by the thymine (T) on the gold film surface of fiber optics, forming an Hg^2+^–T complex. Afterwards, the captured Hg(ii) ions interact with the T modified on the surface of the AuNPs, creating a stable sandwich structure of Au/T–Hg^2+^–T/AuNPs. The strong electromagnetic interaction between the gold nanoparticles (AuNPs) and the gold film causes a change in the resonance wavelength of SPR. The developed sensor had an impressive sensitivity to Hg(ii) in the 80 nM–20 μM range, with a 9.98 nM limit of detection. Moreover, it was successfully applied to Hg(ii) detection in real environmental samples, producing exceptional recovery rates.^77^ Similarly, by combining the sensitive Tyndall effect (TE) of colloidal gold nanoparticles (AuNPs) with particular thymine–Hg^2+^–thymine (T–Hg^2+^–T) coordination chemistry, Xuejiang Chen *et al.* developed a colorimetric approach for Hg(ii) ions detection. The Tyndall effect-inspired assay (TEA) involves the selective hybridization of three types of flexible single-stranded DNAs (ssDNAs) made possible by the presence of Hg(ii) in a sample. This hybridization results in the production of stable rigid double-stranded DNAs (dsDNAs) through the T–Hg^2+^–T ligand interaction. Following the self-assembly of the double-stranded DNAs (dsDNAs) with terminal thiol groups on the surfaces of the gold nanoparticles (AuNPs), their aggregation doubles, along with the insufficient presence of single-stranded DNAs (ssDNAs) as stabilizing agents in a high-salt solution. As a result, there is a noticeable increase in the Tyndall effect (TE) signal, which is proportionate to the Hg(ii) level. The results show that this TE-inspired assay (TEA) approach, which uses a cheap handheld laser pointer pen as a light source to elicit the TE response, enables rapid visual qualitative analysis of 25 nM Hg(ii) in less than 10 minutes. Additionally, using a smartphone to do portable TE measurement makes it easier to quantitatively detect Hg(ii) ions throughout a linear concentration range of 156 to 2500 nM, with a LOD of 25 nM.^78^ Hsin-Yun Chang *et al.* devised a fluorescent sensor labeled as BSA@R6G/MPA-Au NP *via* introducing bovine serum albumin (BSA) to a solution of AuNPs containing rhodamine 6G (R6G) and 3-mercaptopropionic acid (MPA) as depicted in Scheme 3. BSA used to protect R6G/MPA-AuNPs from settled aggregation in high salt concentration. The NPs works as sensor since mercury ions deposited on the surfaces of the AuNPs induce the release of R6G molecules into solution and thus restore the fluorescence of R6G. So, prepared BSA@R6G/MPA-Au NP probe demonstrated the ability to sense mercury ions under high salt conditions. The detection mechanism for mercury involved the deposition of mercury species onto the surfaces of the AuNPs, resulting in the release of R6G molecules into the solution, thereby increasing the fluorescence intensity of the BSA@R6G/MPA-AuNP solution. The specificity of this nanosensor system towards total organic mercury in comparison to Hg(ii) was notably high, with a detection limit of 10 nM and a selectivity of 100-fold.^79^

Desai *et al.* concurrently detected four divalent metal ions Hg(ii), Cu(ii), Pb(ii), and Cd(ii) using a nanosensor based on La^3+^ ions and bovine serum albumin gold nanoclusters (La^3+^ ion–BSA-AuNCs). Fluorescence amplification was found in BSA-AuNCs upon the addition of La^3+^ ions, exhibiting an emission peak at 652 nm under excitation at 323 nm. The resulting nanosensor functioned as a fluorescent sensor for the detection of the four divalent metal ions through mechanisms involving fluorescence quenching (Hg(ii), Cu(ii), and Pb(ii)) and fluorescence enhancement (Cd(ii)). Specifically, for Hg(ii) ions, the emission intensity of La^3+^ ion–BSA-AuNCs decreased attributed to the creation of Au–Hg nanoaggregates through d^10^–d^10^ interactions. The fluorescent nanosensor based on La^3+^ ion–BSA-AuNCs demonstrated good linearity, with LOD of 0.02, 0.048, 0.19, and 4.93 mM for Hg(ii), Cu(ii), Pb(ii), and Cd(ii) ions, correspondingly.^80^ Detection in mM concentration with instant fluorometric response make promising sensor to developed Au-based nano sensor for mercury sensing in real applications.

A sophisticated sensing platform was developed utilizing a nanohybrid composed of silicon particles/gold nanoclusters (SiNPs/AuNCs) to detect Hg(ii) and cysteine through an “on–off–on” switch mechanism. Within this platform, SiNPs played the role of an internal reference signal, contributed inherently correction for background interferences and environmental factors. The AuNCs, covalently attached to SiNPs *via* an amidation reaction, functioned as the reporting unit for detecting Hg(ii). Upon the introduction of Hg(ii), the fluorescence intensity of SiNPs/AuNCs was significantly quenched, resulting in a distinguishable change in fluorescent color. The devised nanoprobe showcased a clear linear correlation between the ratiometric fluorescence signal (*F*_649_/*F*_511_) and the concentration of Hg(ii), spanning from 0.02 to 24 mM, and achieving an impressive LOD of 5.6 nM. The achieved detection limit significantly far below the WHO-recommended guideline value for Hg(ii) in drinking water. The fluorescence sensing process likely involves the dispersion of Hg(ii) around the AuNCs due to the notable affinity between Hg(ii) and AuNCs. However, redox cannot occur in the system, thus the Hg(ii) on the surface of the AuNCs cannot create a non-fluorescent gold amalgamation. The ratiometric probe's Hg(ii) quenching mechanism is most likely generated by a strong and specific d^10^–d^10^ contact between Hg(ii) (4f^14^–5d^10^) and the coated Au^+^ (4f^14^–5d^10^), known as the metallophilic effect.^81^ A label-free colorimetric Hg(ii) nanosensor have developed by exploiting the inhibitory effect of Hg(ii) on the kinetic aspect of the growth of gold nanoparticles on the surface of gold nanostars (AuNS). The H–AuNS probes were modified using 2-[4-(2-hydroxyether)piperazin-1-yl]ethanesulfonic acid (HEPES). Following thorough reagent and experimental condition optimization, the HEPES-coated AuNSs (H–AuNSs) demonstrated excellent selectivity and sensitivity in detecting Hg(ii). The H–AuNS probe detected Hg(ii) in HCl/Au(iii)/H_2_O_2_ at concentrations ranging from 1.0 nM to 100 μM, with an outstanding LOD of 0.7 nM. Furthermore, the optical detection limit was revealed to be 10 nM, allowing for easy detection with the naked eye in real samples.^82^

Chang *et al.* developed gold nanodots (11-MUA-Au ND) protected with 11-mercaptoundecanoic acid (11-MUA) for mercury detection. The Au NDs@11 MUA probe effectively detects the total concentration of mercury ions, encompassing both inorganic and organic mercury species, within an aqueous solution. The nanosensor system shows remarkable selectivity for total mercury over other metal ions, with 1000 times higher sensitivity and a LOD of 2.0 nM. This sensitive and selective technology has long-term practical promise when compared to traditional ways for therapeutically assessing mercury ions in biological fluids.^83^ Furthermore, cysteine-modified and glutathione-stabilized indium-based organometallic nanoclusters/structures (AuNCs/MIL-68(In)–NH_2_/Cys) have developed for Hg(ii) detection. The nanosensors exhibit vibrant pink fluorescence with AuNCs uniformly dispersed on MIL-68(In)–NH_2_. Under excitation at 370 nm, the sensor demonstrates dual fluorescence emissions at approximately 438 nm and 668 nm, corresponding to MIL-68(In)–NH_2_ and GSH–AuNC, respectively. The fluorescence emission experienced a notable enhancement following the modification with Cys. When Hg(ii) is present, the blue fluorescence peak at 438 nm changes somewhat while the red fluorescence peak at 668 nm becomes less intense. The primary mechanism responsible for quenching fluorescence is the impact of heavy metal ions on the interaction between Au^+^ (4f^14^–5d^10^) and Hg(ii) (4f^14^–5d^10^). With a LOD of 6.7 pM, the produced AuNCs/MIL-68(In)–NH_2_/Cys nanosensor has two linear Hg(ii) detection ranges: from 20 pM to 0.2 μM and from 0.2 μM to 60 μM. Moreover, a radial paper microfluidic analyzer (μPAD) in a star-shaped configuration was effectively constructed, providing a straightforward and convenient platform for visually detecting Hg(ii) across a broad detection range spanning from 5 nM to 50 μM.^84^

**Scheme 4:**
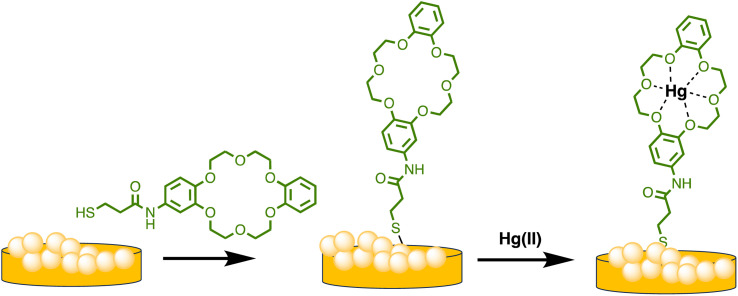
Graphic for nanostructured Au substrate modification by TCE and Hg(ii) ions binding.

Similarly, Sarfo *et al.* developed a poly-thymine (T) aptamer/2-naphthalenethiol (2-NT) modified AuNPs as a SERS sensor for Hg(ii) sensing. 2-NT serves as a Raman reporter, and the T aptamer can form a T–Hg(ii)–T structure with Hg(ii) ions, allowing for the absorption of the SERS nanosensor onto the SERS chip. This nanosensor exhibits a LOD of 1.0 ppt (1.0 × 10^−12^ g mL^−1^), which is much lower than the WHO's recommended drinking water level of 10.0 ppb.^85^ Similarly, as illustrated in Scheme 4, Sarfo and Sivanesan *et al.* devised a SERS sensor by conjugating aminodibenzo-18-crown-6 with mercaptopropionic acid. The resulting crown ether derivative (TCE) self-assembled on the surface of the gold nanostructure of the substrate to form a distinct surface layer for Hg(ii) ions. The interaction between Hg(ii) and the oxygen atoms of the TCE resulted in the automatic binding of the metal ion within the cavity of the crown ether layer. As a consequence, there was a rise in the intensity of the Raman band at 1501 cm^−1^ for crown ethers with a Hg(ii) concentration ranging from 1 × 10^−11^ M to 1 × 10^−6^ M. The threshold value of Hg(ii) was quantitatively determined using the new SERS method. Nanosensor and the detection limit was 1000 times lower than the values for Hg(ii) ions in water reported by EPA and WHO.^86^

**Fig. 11:**
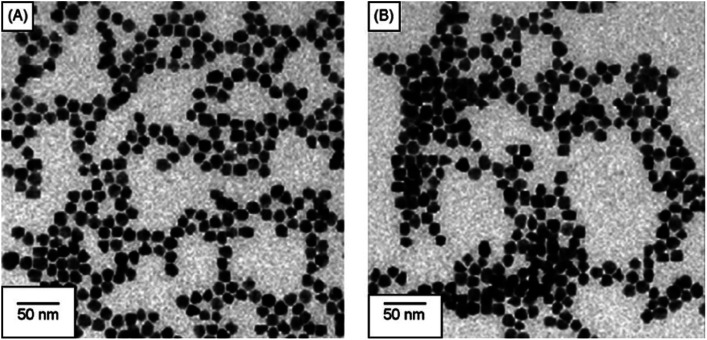
(A) TEM of Rh6G–TGA@AuNPs without Hg(II) and (B) with Hg(ii) ion (reproduced from ref. 87 under the license of freely access to reuse).

Similarly, Chen *et al.* produced a fluorescent sensor that detects Hg(ii) in aqueous solution using gold nanoparticles (AuNPs) and rhodamine 6G (Rh6G).^87^ AuNPs were prepared and modified with thioglycolic acid (TGA). In bulk solution, free rhodamine 6G (Rh6G) dye exhibited strong fluorescence. However, when Rh6G was combined with AuNPs, the resulting sensor system showed weak fluorescence due to FRET and collision effects. When Hg(ii) was present, Rh6G units separated from the surface of functionalized AuNPs, causing the fluorescence of the AuNPs-based sensor to gradually recover. Further, morphological changes in NPs after the addition of Hg(ii) ions also examined by TEM analysis. Fig. 11A demonstrates that before adding Hg(ii) to the AuNPs solution, the AuNPs are regular and near-spherical, appearing monodisperse with an average size of 13.3 ± 1.2 nm. Whereas as in Fig. 11B depicted after addition of Hg(ii), a slight aggregation of AuNPs observed that was driven by Hg(ii) ions. Under optimized conditions, the fluorescence intensity of the sensor correlates with the concentration of Hg(ii). Calibration curves exhibit linearity within the range of 5.0 × 10^−10^ to 3.55 × 10^−8^ mol L^−1^, with a LOD of 6.0 × 10^−11^ mol L^−1^.

**Fig. 12:**
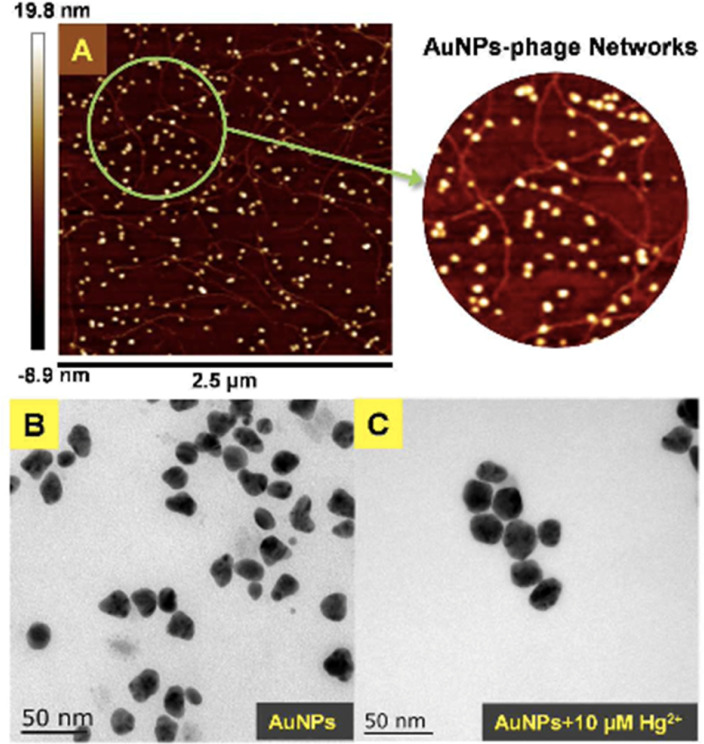
(A) AFM image and (B and C) TEM images of AuNPs-phage network without and with the addition of Hg(ii) (reproduced from ref. 89 with permission from Royal Society of Chemistry, Copyright @ 2017).

Moreover, based on the analyte-induced etching and amalgamation of AuNPs, an innovative colorimetric headspace nanosensor was devised for the specific detection of Hg(ii) ions. Initially, Hg(ii) underwent reduction to its volatile form Hg(0) facilitated by SnCl_2_*via* a cold chemical vaporization reaction. Hg(0) was subsequently extracted into a 37 μL aqueous suspension of thioglycolic acid-functionalized AuNPs with 10% methanol serving as an extractant. Simultaneously, it underwent a reaction with AuNPs through a robust metallophilic Hg–Au interaction, leading to a color change from red to blue. The LOD values were determined to be 5 nM by naked eyes and 1 nM by UV-visible measurement, which is below the safe limit of Hg(ii) in drinking water established by the US EPA. This demonstrates significant possibilities for monitoring extremely low levels of Hg(ii) in ambient water samples.^88^ Gold nanoparticles (AuNPs) were synthesized *in situ* on the surface of M13 phages that bind Hg(ii) at room temperature. The obtained AuNP phase networks were subsequently utilized for the direct detection of mercury. Hg(ii) selectively bound to the M13 phages situated on the networks and gathered around the AuNPs. This was followed by reduction to Hg(0) and deposition onto the surface of the AuNPs, inducing a blue shift in the spectral properties. For practical applications, “purple” AuNP phase gratings with an SPR absorption peak at 550 nm were selected for Hg(ii) detection due to their better response compared to “pink” AuNP phase gratings with a range of 518 to 538 nm. The AuNP phase networks changed from light purple to deep pink with a shift to blue and an increase in SPR upon addition of Hg(ii). These observations were further supported by the TEM analysis, as shown in Fig. 12, where the “purple” AuNPs were exhibited well dispersion with an average diameter of 20–30 nm. The discrepancy between the SPR peak and the particle size was attributed to the unequal triangular shape of the AuNPs rather than particle aggregation. Subsequently, the AuNPs became rounder and larger after the addition of Hg(ii). The morphological changes of the AuNPs were a consequence of the reduction of the AuCl_4_ residue in the solutions of the AuNP phase network or the deposition of Hg(0) on the edges of the triangular AuNPs. The Hg(ii) observed in this way was initially captured by the phases and reduced to Hg(0) by the reducing functional groups on the phase surfaces. Conversely, owing to the robust affinity between Hg and Au, Hg(0) can further deposit on the gold surface, leading to the formation of an Au–Hg alloy.^89^

Recently, Chen *et al.* introduced an innovative colorimetric nanosensing approach for Hg speciation, utilizing the analyte-induced aggregation of AuNPs coupled with a thiol-containing diethyldithiocarbamate (DDTC) ligand. Since Hg–DDTC was more stable than Cu–DDTC, when mercury species were added, a location shift occurred between the Hg and Cu(ii) species, causing the functionalized Au nanoparticles to aggregate and cause a color change. In addition, due to the masking effect of EDTA, the nanosensor can easily distinguish organic mercury from inorganic mercury (Hg(ii)) and is therefore expected to be the case sheds light on the colorimetric detection of organic mercury. In this manner, a straightforward and easy colorimetric test for the identification of Hg species was produced, which is characterized by high detectability, for example up to 10 nM for Hg(ii) and 15 nM for methylmercury.^90^ An comparative overview of Au based nanosensor given in Table 3.

**Table 3:** An overview of gold-based nano-sensors for sensing of mercury metal ion

Entry	Nano sensor	Sensing approach	Linear range	LOD	Ref.
1	AuNPs	Colorimetric and optical	—	20 ppb	76
2	AuNPs/T	SPR	80–20 μM	9.98 nM	77
3	Thymine@AuNPs	Tyndall effect	156 to 2500 nM	25 nM	78
4	BSA@R6G/MPA–Au NP	Fluorescent	—	10 nM	79
5	La^3+^ ion–BSA-AuNCs	Fluorescent	—	0.02 mM	80
6	SiNPs/AuNCs	Fluorescent	0.02 to 24 mM	5.6 nM	81
7	HEPES-capped AuNSs	Colorimetric	1.0 nM–100 μM	0.7 nM	82
8	11-MUA-Au NDs	Colorimetric	—	2.0 nM	83
9	AuNCs/MIL-68(In)–NH_2_/Cys	Fluorescent	20 pM to 0.2 μM	6.7 pM	84
10	Poly-thymine(T)aptamer/2-naphthalenethiol(2-NT) modified AuNPs	SERS	—	1.0 ppt	85
11	Crown ether@AuNPs	SERS	1 × 10^−11^ M to 1 × 10^−6^ M	—	86
12	(AuNPs)–rhodamine 6G (Rh6G)	Fluorescent	5.0 × 10^−10^ to 3.55 × 10^−8^ mol L^−1^	6.0 × 10^−11^ mol L^−1^	87
13	Thioglycolic acid functionalized AuNP	Colorimetric	—	5 nM	88
14	AuNPs-phase networks	Redox	—	8 × 10^−8^ mol L^−1^	89
15	DDTC@AuNPs	Ion displacement	—	10 nM	90

### 2.3. Ag–Au hybrid nano-sensor for mercury sensing

**Scheme 5:**
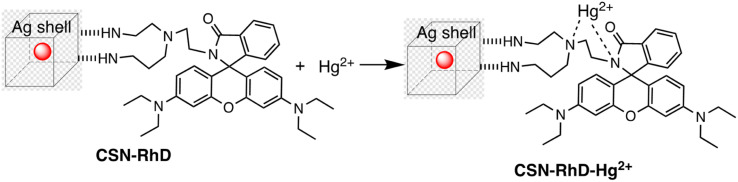
Binding mode of CSN-RhD for Hg(ii) metal ion sensing.

Ag–Au hybrid nano sensors represent promising approach for the mercury sensing, capitalizing on the synergistic properties of silver (Ag) and gold (Au) nanoparticles. By combining the unique characteristics of these two noble metals, such as the excellent conductivity of silver and the remarkable stability of gold, these hybrid nano sensors offer enhanced performance in terms of sensitivity and selectivity for detecting mercury ions. Further, Ag–Au interface provides a versatile platform for functionalization, allowing for tailored surface modifications that improve the sensors' affinity towards mercury. The synergistic relationship between the optical properties of gold and the catalytic property of silver enables dual-mode detection, utilizing both colorimetric and electrochemical signals for robust and multifaceted mercury detection. For example, Yue Wang and co-workers have produced Janus nanoparticles, a hybrid nanomaterial comprising gold nanorods (AuNR) coated with silver (Ag) and polyaniline (PANI) for mercury detection. The hybrid material's morphology and structural characteristics are defined by the synergistic effects of the optical and electrical properties of organics (PANI) and the LSPR properties of Au and AgNPs. These properties are critical for sensing. So, (AuNR@Ag)-PANI NPs were fabricated using a droplet-based microfluidic technique, ensuring excellent dispersion and a consistent structure. Excellent affinity response to Hg(ii) ions and SERS activity are displayed by the synthesized (AuNR@Ag)-PANI JNPs. The method for detecting Hg(ii) relies on the coordination interaction between the nitrogen atoms containing lone pairs of electrons within PANI in the (AuNR@Ag)-PANI JNPs and the Hg(ii) ions. The Raman intensity of PANI experienced augmentation due to its significant binding affinity with Hg(ii) ions. The detection limit for Hg ion concentration was determined to be 0.97 nM, with a good linear correlation observed between the increases in Raman intensity of PANI and the concentration of Hg(ii) ions within the range of 1–150 nM.^91^ Comparably, a dual-mode sensor that could analyze mercury(ii) using both metal-enhanced fluorescence (MEF) and SERS was produced for the rapid detection of mercury. The CSN-RhD sensor is made up of size-dependent core–shell nanocubes (CSN), that were made of gold nanospheres (AuNS) coupled with rhodamine derivatives (RhD) and covered with a layer of silver. It was demonstrated that when the thickness of the Ag cubic shell increased, the SERS activity of the CSN with a spherical core increased. Under ideal circumstances, strong MEF and SERS signals of the resulting mixtures with rising Hg(ii) concentrations were observed. With a broad linear range of 0.001–1000 ppm and 0.01–1000 ppm, as well as a LOD of 5.16 ppb for SERS tests and 0.94 ppb for MEF assays, the suggested bimodal sensor demonstrated exceptional performance for Hg(ii). Scheme 5 illustrates the unique detection mechanism of the bimodal CSN-RhD sensor. In MEF assays, Hg(ii) binds to RhD's lone electron pair, causing the spirolactam ring-opening process of RhD. This leads to the formation of a fluorescence emissive complex, which causes the photoinduced process to “switch off.” In contrast, for SERS assays, CSN-RhD was conjugated to the Hg(ii) surfaces, displaying a strong electron transfer (PET) process and a corresponding increase in fluorescence intensity. Additionally, the sensor's capacity to identify Hg(ii) in tampered milk samples was verified.^92^

Further, in the development of a silver–gold alloy sensor, the SERS enhancement property of 4-aminothiophenol (4-ATP) as a signal turn-off method has been utilized. This sensor, termed 4-ATP (Ag–Au/4-ATP), demonstrates effective sensing capabilities towards Hg(ii) through a SERS off-signal. Different chemometric algorithms were utilized to analyze the obtained SERS and ICP-MS chemical reference data, aiming to identify optimal wavelengths and spectral variables for constructing models to detect Hg(ii) in both standard solutions and spiked tea samples. Significantly below the threshold level of 0.5 mg kg^−1^ in food, the LOD was found to be 4.12 × 10^−7^ μg mL^−1^ for Hg(ii) in standard solutions and 2.83 × 10^−5^ μg g^−1^ for Hg(ii) in spiked tea samples. With relative standard deviations of 1.14% and 0.84%, sensor exhibited high stability and reproducibility. The significant correlation noted between the SERS sensor and the chemical reference technique underscores the promise of the chemometrics-integrated SERS system established for future investigation and assessment of Hg(ii) concentrations in tea.^93^ An comparative overview of Ag–Au hybrid based nanosensor presented in Table 4.

**Table 4:** An overview of Ag–Au hybrid nano-sensors for sensing of mercury metal ion

Entry	Nano sensor	Sensing approach	Linear range	LOD	Ref.
1	(AuNR@Ag)-PANI JNPs	SERS	1–150 nM	0.97 nM	91
2	CSN-RhD	MEF and SERS	MEF = 0.001–1000 ppm and SERS = 0.01–1000 ppm	MEF = 0.94 and SERS = 5.16 ppb	92
3	4-ATP (Ag–Au/4-ATP) SERS	SERS off-signal	0.01–0.00001 μg mL^−1^	For standard solution 4.12 × 10^−7^ μg mL^−1^ and 2.83 × 10^−5^ μg g^−1^ for spiked tea samples	93

### 2.4. Carbon dot-based nano-sensor for mercury sensing

**Scheme 6:**
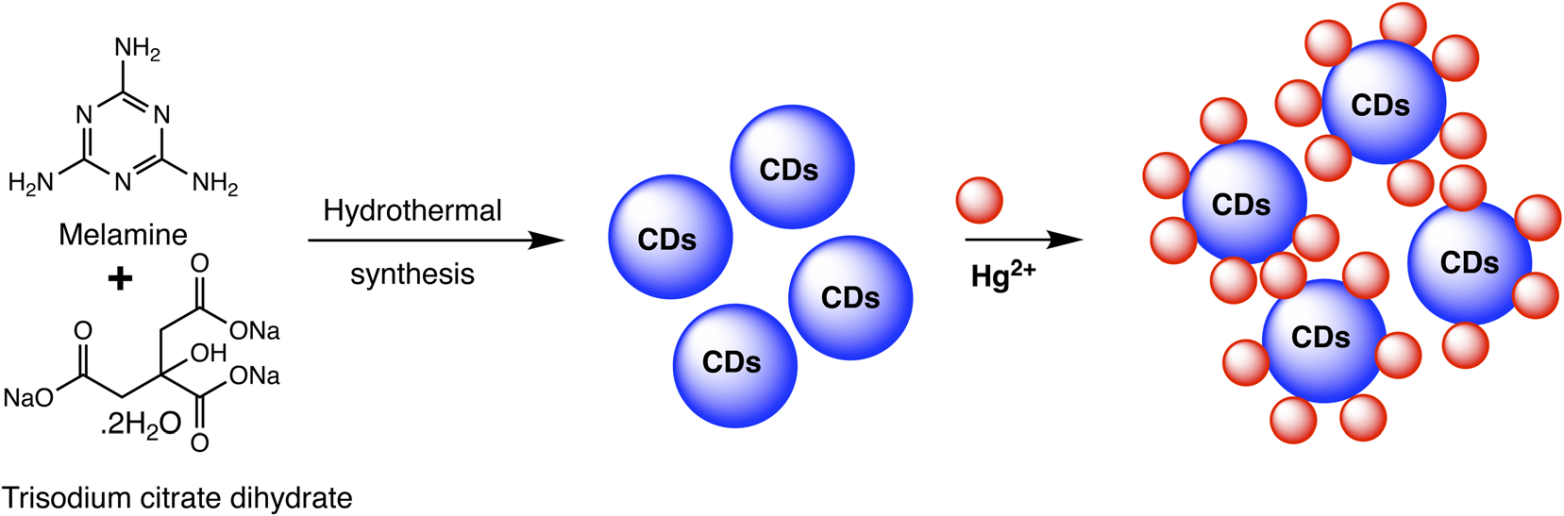
Schematic of CDs preparation using melamine and trisodium citrate dehydrate precursor and Hg(ii) detection.

Carbon dots (CDs) are typically carbonaceous nanoparticles with dimensions typically smaller than 10 nanometers. Due to their unique optical, electronic, and chemical properties, CDs significantly serve as sensing platforms for detection of various analytes.^94^ By utilizing the special qualities of carbon dots to detect mercury ions with extreme sensitivity and selectivity, CD-based nanosensors provide an intriguing new direction in the field of mercury detection. These nanosensors can be finely tuned for mercury detection through surface functionalization and chemical modifications. Such as the fluorescent CDs developed by Cheng *et al.* to sense Hg(ii), as illustrated in Scheme 6. In a typical synthetic protocol, the precursors (melamine and trisodium citrate dehydrate) are carbonized together at high temperatures. The final CDs had a bright blue luminescence after that. Upon addition of Hg(ii) to CDs solution, the Hg(ii) ions led to complexation with CDs *via* interaction with carboxyl groups that are present on CDs surface. The luminous CDs aggregate because of this interaction. It implies that the higher Hg(ii) concentration may extinguish the CDs' fluorescence. As nanosensors for the detection of Hg(ii), CDs made in accordance with this technique were employed. The blue fluorescent CDs that were made show remarkable stability along with good selectivity and sensitivity when used to probe Hg(ii) by fluorescence quenching at a concentration of 15 nM. Consequently, there is a lot of promise for the quick analysis of Hg(ii) in ambient material using the new fluorescent CDs.^95^

**Scheme 7:**
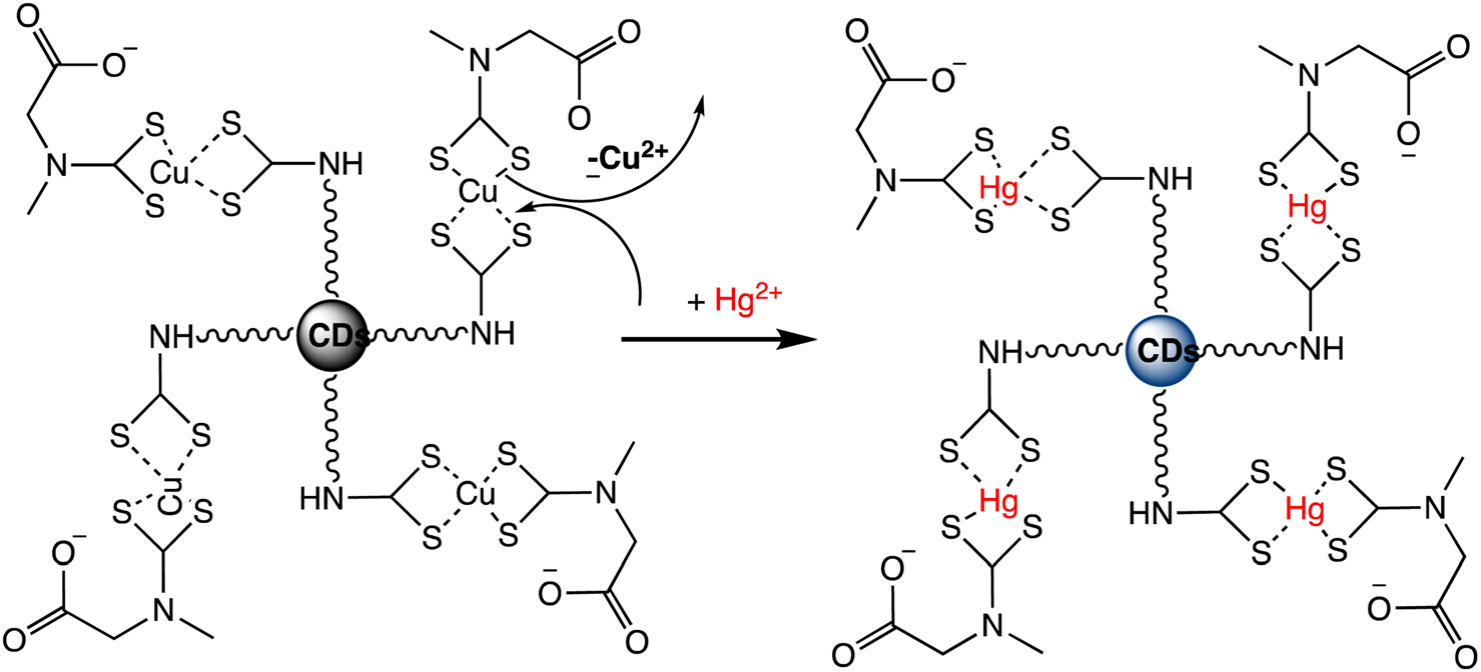
Fluorescent “Turn On” nanosensor for surface-bound CuDTC2 complex Hg(ii) detection based on Cu(ii) displacement due to Hg(ii).

A novel “turn-on” fluorescence nanosensor has been developed to selectively detect Hg(ii) ions. This innovative sensor utilizes carbon nanodots that have been modified with bis-(dithiocarbamato)copper(ii) (CuDTC2-CDs). As carbon disulfide condensed onto nitrogen atoms found in the surface amine groups, the CuDTC2 complex was linked to the amine-coated CDs. CuDTC2-complexing CDs were subsequently formed as a result of copper(ii) bonding with the ensuing dithiocarbamate groups (DTC) and ammonium *N*-(dithicarbaxy) sarcosine (DTCS) coordinating. CuDTC2-CDs showed a notable reduction in their bright blue fluorescence, which was attributed to a combination of energy and electron transfer pathways. Further, upon the addition of Hg(ii) ions, the fluorescence of CuDTC2-CDs was rapidly activated as it displaced Cu(ii) ions within the CuDTC2 complex, thus interrupting the energy transfer pathway. This process is depicted in Scheme 7. Importantly, this nanosensor displayed high sensitivity for detecting Hg(ii), with a LOD as low as 4 ppb.^96^ In a similar approach, a binary sensing strategy has been successfully validated for the highly sensitive and selective detection of Hg(ii) and l-cysteine (l-Cys), utilizing water-soluble carbon dots (CDs) as an innovative fluorescent probe. Mercury ions can effectively interact with the surface of CDs through electrostatic forces. ‘Turn-off’ effect: this causes a dramatic decrease in the fluorescence intensity of the CDs *via* fluorescence charge transfer. Moreover, the CDs–Hg complex exhibits sensitivity towards l-Cys due to its ability to form strong Hg–SR bonds. Subsequently, with the addition of l-Cys, Hg(ii) ions preferentially bind to l-Cys rather than to CDs, causing the removal of Hg from the CDs' surface and subsequently shielding fluorescence quenching. As a result, a significant enhancement in the fluorescence of the CDs is observed (turn-on). Under optimized conditions, a satisfactory linear range for Hg sensing from 2 to 22 μM has been attained, with a LOD of 0.017 μM.^97^ Correia *et al.* synthesized Eu–Cdots using a hydrothermal method, where citric acid and urea served as precursor materials, and Eu(NO_3_)_3_ was utilized as the europium source. The Eu^3+^ cation interacted with functional groups present on the surface of the C-dots, thereby incorporating into the carbon network and forming Eu–O charge transfer complexes. These Eu–Cdots functioned as luminescent sensors for Hg(ii) and Ag(i) cations, resulting in a decrease in luminescence intensity observed in aqueous solutions within the concentration range of 10–100 μM. The LOD for Hg(ii) was determined to be 4–5 μM. Hg(ii) and Ag(i) ions effectively suppress the broad emission of C-dots at 450 nm, along with the distinct emission bands of Eu^3+^ ions. Specifically, Hg(ii) and Ag(i) ions induce a significant decrease in the luminescence intensity of C-dots, while other cations demonstrate minimal influence. The quenching mechanism exhibits variability based on the ion's specific characteristics. The presence of Hg(ii) results in the suppression of both the blue emission of C-dots and the red emission of Eu^3+^, whereas the presence of Ag(i) solely impacts the emission of C-dots.^98^

**Scheme 8:**
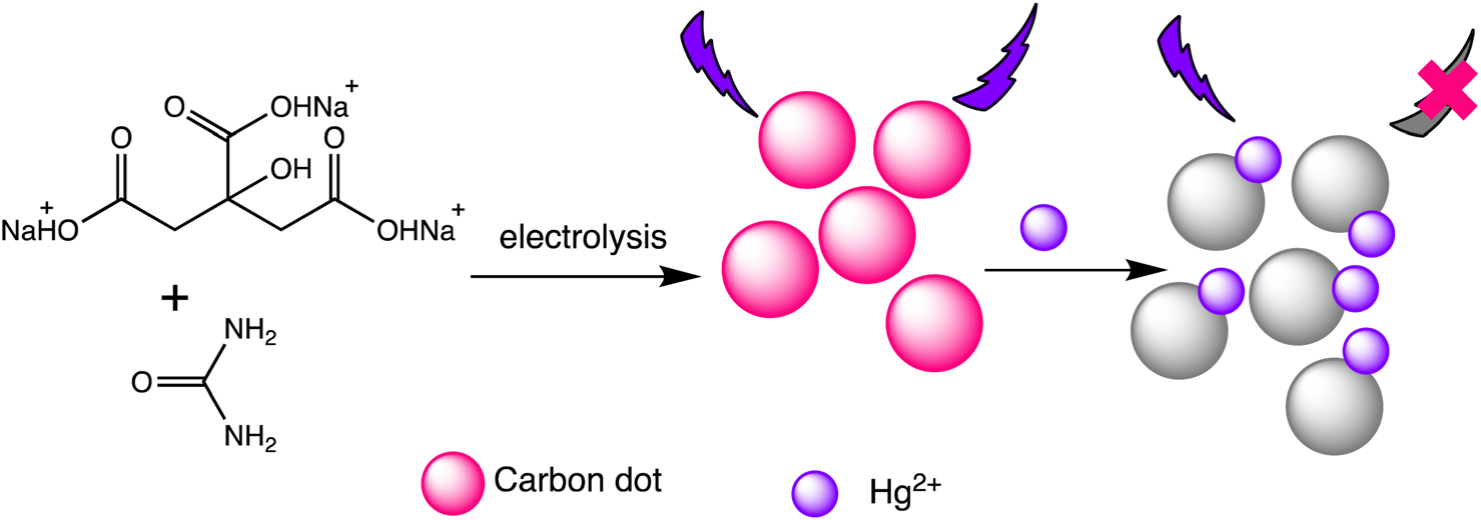
Graphic for Hg(ii) and Cys detection *via* the carbon dots.

Liu *et al.* have developed two new ratio-metric fluorescent nanosensors utilizing multi-emission CD nanohybrids for the specific and selective recognition of Pb(ii) and Hg(ii) ions. Nanohybrids were prepared through a single-step green solvothermal treatment of natural biomass extracted from bamboo leaves. This strategy proves highly advantageous since it avoids the requirement for CDs to be post-modified or their combination with other fluorescent nanomaterials. Impressively LODs were attained for Pb(ii) (0.14 nM) and Hg(ii) (0.22 nM) ions utilizing the dual- and three-emission CD nanohybrids prepared in this study, respectively. The WHO's suggested acceptable levels for the two heavy metal ions in drinking water (Pb(ii): 48 nM and Hg(ii): 5 nM) are noticeably lower than these LODs. Furthermore, fluorescence spectra analysis revealed that the fluorescence intensity at 611 nm decreases with increasing Hg(ii) concentration, demonstrating clear negative ion level-dependent responses. Surprisingly, a red shift behavior was noticed alongside the reduction in fluorescence, which is likely attributable to the formation of a larger conjugation system once the porphyrin compounds on the nanohybrids bind Hg(ii) ions.^99^ Helena Gonçalves *et al.* developed carbon nanoparticles (CNPs) through the direct laser ablation of carbon targets immersed in water. The laser ablation parameters were precisely adjusted to generate carbon nanoparticles with diameters of up to 100 nm. After functionalization with *N*-acetyl-l-cysteine (NAC) and NH_2_-polyethylene glycol (PEG200), the carbon nanoparticles showed fluorescence peaks at wavelengths of 450 nm and 340 nm, respectively. When Hg(ii) and Cu(ii) ions are present, the fluorescence intensity of the NPs decreases, with Stern–Volmer values of 1.3 × 10^5^ and 5.6 × 10^4^ M^−1^, respectively.^100^ Furthermore, Lu and Wu *et al.* proposed a novel turn-on ratiometric fluorescence assay for glutathione (GSH) sensing, using carbon dots (CDs) and rhodamine B (RhB), based on the recovered luminous intensity of the CDs–Hg(ii) combination. When excited at a wavelength of 350 nm, the nanohybrid system displayed two emission peaks at 440 nm and 570 nm. The fluorescence emitted by the functional CDs was diminished as a result of electron transfer occurring between the CDs and Hg(ii). With the addition of GSH, the fluorescence of the CDs–Hg(ii) system showed gradual recovery, attributed to the selective bonding of GSH to Hg(ii) *via* Hg–S bonding interactions, while the fluorescence of RhB remained unaffected. A LOD of 25 nM for Hg(ii) was achieved, demonstrating a robust linear relationship between the I440/I570 ratios and the concentrations of Hg(ii) ranging from 0.5 to 10 μM.^101^ Water-soluble functionalized fluorescent C-dots were synthesized as depicted in Scheme 8 through the electrochemical carbonization of sodium citrate and urea. The C-dots primarily range in size from 1.0 to 3.5 nm, with approximate size of 2.4 nm. These C-dots demonstrate exceptional photostability and possess a high quantum yield of 11.9%. The fluorescence intensity of the C-dots at 433 nm gradually decreases with increasing concentrations of Hg(ii). They offer selective detection of Hg(ii) ions within a linear detection range from 0.01 to 10 mM, with a LOD of 3.3 nM.^102^

**Scheme 9:**
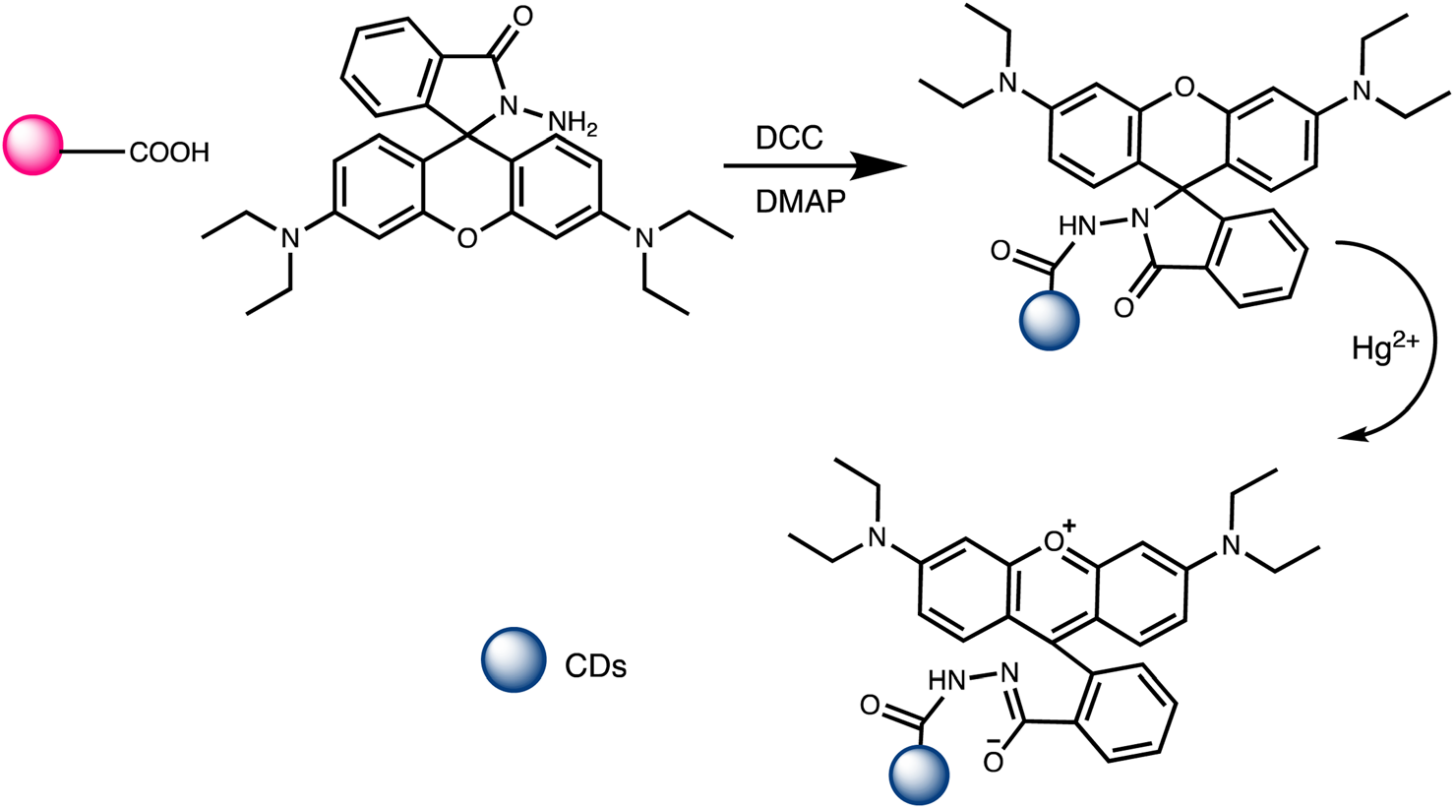
Schematic representation for RbH-CDs preparation and Hg(ii) detection.

Ma and Mei *et al.* developed a ratiometric nanosensor for Hg(ii) utilizing rhodamine B hydrazide-functionalized carbon dots (RbH-CDs). These composite nanosensors comprise blue-fluorescent CDs synthesized from citric acid monohydrate *via* a one-step heating method. The RbH-CDs nanosensor exhibits a distinct blue fluorescence at 450 nm, corresponding to the fluorescence peak of the CDs. However, upon exposure to Hg(ii), the rhodamine moieties undergo a ring-opening process, leading to a substantial increase in fluorescence emission at 575 nm from the rhodamine molecules. This enhanced emission enables a ratiometric fluorescence response for Hg(ii) detection. When Hg(ii) is added, the CDs-RbH nanohybrid solution's fluorescence progressively changes from blue to orange during the sensing process, facilitating easy observation with the naked eye under UV irradiation. Moreover, the CDs-RbH nanohybrid system exhibits notable sensitivity towards Hg(ii) owing to the collective influence of the two fluorescent compounds, along with enhanced selectivity towards Hg(ii) compared to other competing metal ions. A possible sensing mechanism is illustrated in Scheme 9. Upon the addition of mercury ions, the open-ring configuration of the rhodamine unit attached to the surface of the CDs undergoes a noticeable enhancement in the orange emission of the open-ring rhodamine, which correlates with the concentration of Hg(ii) ions. Consequently, distinct dual emissions are observed at 450 nm and 575 nm when excited with a single wavelength of 360 nm.^103^ Recently, Samota *et al.* reported carbon dots doped with sulfur and nitrogen (N, S-CDs) for mercury detection, utilizing Typa angustata Bory as a precursor. The quantum yield of the N, S-CDs was high and were significantly quenched in the presence of Hg(ii) ions without interference from other interfering ions. Due to their extraordinary quantum yield, a linear detection range of 0.01–60 μM was achieved, with a LOD of 3.1 nM for Hg(ii).^104^ An comparative overview of carbon dot based nanosensor presented in Table 5.

**Table 5:** An overview of carbon dot-based nano-sensors for sensing of mercury metal ion

Entry	Nano sensor	Sensing mechanism	Linear range	LOD	Ref.
1	CDs	Fluorescent	—	15 nM	95
2	CuDTC2-CDs	Fluorescent	—	4 ppb	96
3	Water-soluble CDs	Fluorescent	2 to 22 μM	0.017 μM	97
4	Eu–Cdots	Fluorescent	10–100 μM	4–5 μM	98
5	CD nanohybrids	Fluorescent	—	0.22 nM	99
6	RhB@CDs	Fluorescent	0.5 to 10 μM	25 nM	101
7	RbH-CDs	Fluorescent	0–100 μM	—	103
8	N, S-CDs	Fluorescent	0.01–60 μM	3.1 nM	104

### 2.5. Quantum dot-based nano-sensor for mercury

Quantum dots are semiconductor particles (2–10 nm), their size-dependent tunable optical and electronic characteristics, provide a versatile platform for designing sensitive sensors. In the presence of mercury ions, quantum dots exhibit distinct changes in fluorescence, enabling precise and rapid detection. The quantum confinement effect allows for the engineering of quantum dots with specific bandgaps, optimizing their performance for mercury detection applications.^105^ For instance, Gaurav Bhanjana *et al.* have devised ZnO quantum dots as an effective electron mediator for highly sensitive and selective electrochemical detection of Hg(ii) ions. The prepared nanosensor exhibits a remarkably LOD of 5 ppb, with a sensitivity of 4.6 μA cm^−2^ ppm^−1^ and a response time of less than 2 seconds. Moreover, the fabricated sensor demonstrates reproducibility and stability for up to three months.^106^ Similarly, nitrogen and sulfur co-doped graphene quantum dots (N, S-GQDs) were synthesized *via* a single-step hydrothermal synthesis method. This method facilitated the highly sensitive and precise detection of mercury ions at concentrations as low as the nanomolar level in both aqueous solutions and wastewater samples. Prepared N and S-GQDs have a consistent size distribution, with an approximate average particle size of 3.5 nm. Nitrogen doping contributes to an increased quantum yield (41.9%). while the incorporation of sulphur atoms improves the selectivity towards Hg(ii) by facilitating strong coordination interactions. Upon the addition of Hg(ii), there is a proportional decrease in the fluorescence intensity of N, S-GQDs. This leads to a dynamic range spanning four orders of magnitude and a LOD of 0.14 nM in deionized water. These N, S-GQDs nanosensing probes have exhibited effectiveness in analyzing dye wastewater samples and sewage samples alike. They possess a linear detection range from 0.1 to 15 μM and have yielded recovery rates ranging from 96% to 116%.^107^

Similarly, in an investigation, ZnSe/ZnS nanoparticles (NPs) doped with manganese and enveloped with mercaptopropionic acid (MPA) were employed for the precise detection of Hg(ii) ions, demonstrating both specificity and sensitivity. The sensing capability achieved a LOD of 0.1 nM within a dynamic range spanning from 0 to 20 nM. The aggregation of colloidal NPs ensued due to the detachment of the organic passive layer from the NP surface in the presence of Hg(ii) ions. This aggregation phenomenon was primarily driven by the strong interaction between the mercury ions and the thiol(s). This highly sensitive sensor underscored the remarkable efficacy of mercaptopropionic acid (MPA) as a mercury ion receptor.^108^ Similarly, Ming Li and co-workers developed a composite sensor comprising quantum dots (QDs), DNA, and gold nanoparticles (AuNPs) to detect mercury(ii). When Hg(ii) ions are present in an aqueous solution with DNA-conjugated QDs and AuNPs, DNA hybridization is triggered. This process brings the QDs and AuNPs into proximity, facilitating nanometallic surface energy transfer (NSET) from the QDs to the AuNPs, resulting in the quenching of QDs' fluorescence emission. The LOD for this nanosensor was observed 1.2 ppb and 0.4 ppb Hg(ii) in river water and buffer solution, correspondingly.^109^

**Scheme 10:**
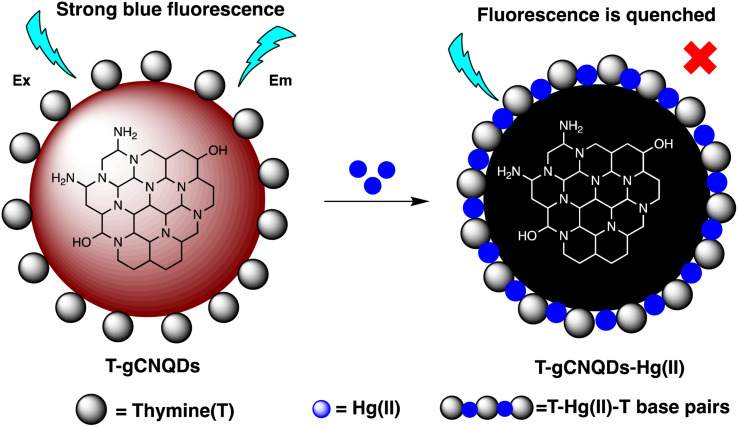
Schematic representation of Hg(ii) binding on T–gCNQDs surface.

M. H. Amini *et al.* have developed functionalized graphene quantum dots (GQDs) as a fluorescent “off–on” nanosensor for mercury and ethyl xanthate detection. The GQDs synthetic protocol involved thermal pyrolysis of citric acid, followed by monoethanolamine (MEA) to functionalize their surfaces. Notable fluorescence emissions with a high quantum yield were the outcome of functionalizing graphene quantum dots (GQDs) with MEA (MEA-GQDs). When Hg(ii) ions are added, the fluorescence emissions of MEA-GQDs are quenched. This is because complexes between the Hg(ii) ions and the functional groups on the MEA-GQDs form. Furthermore, the creation of stronger complexes between the thiol group of EtX- and Hg(ii) ions results in the restoration of the fluorescence intensity when ethyl xanthate (EtX-) ions are present. In optimum conditions, concentration ranges for Hg(ii) and EtX-ions would be 0.05–5 nM and 0.05–3 nM, respectively, with LODs of 10 nM and 30 nM.^110^ Further, Jun Yao and Xin Gou have developed a green, highly luminescent CdTe/CdS for mercury detection. The sensor has high sensitivity, selectivity, and fast response time in a wide linear range with a low detection limit. The LOD is at least 1.7 × 10^−9^ mol L^−1^, which is a lower LOD than most other mercury detection methods.^111^ A novel nanosensor called T–gCNQDs *via* thymine–modified graphitic carbon nitride quantum dots has been devised by Achadu and Revaprasadu *et al.* This fluorescent nanoprobe demonstrates enhanced photoluminescence characteristics due to the incorporation of thymine. When exposed to Hg(ii), a significant decrease in fluorescence occurs, indicating a strong quenching effect resulting from the specific binding between Hg(ii) and the thymine group. The exact contact and binding affinity between Hg(ii) and thymine groups on the surface of T–gCNQDs are shown in Scheme 10, which results in the formation of a non-radiative T–Hg(ii)–T complex. Fluorescence intensity, best measured at excitation and emission wavelengths of 350/445 nm, experiences a notably greater quenching effect by Hg(ii) compared to nanoprobe lacking thymine. This quenching effect, which is linked to the creation of T–Hg(ii)–T base complexes, keeps the selectivity even in the presence of other metal ions. The fluorescence decreases linearly within the Hg(ii) concentration range of 1.0–500 nM, with a LOD of 0.15 nM.^112^

To achieve precise and selective detection of Hg(ii) and cysteine (Cys), Ting Liu and co-workers have established an magnesium–nitrogen-doped highly fluorescent carbon quantum dots Mg–N-CQDs *via* a hydrothermal method. After the addition of Hg(ii), there is a significant reduction observed in the fluorescence of the Mg–N-CQD. This decrease is assigned to the electron transfer mechanism occurring from the surface excited states of Mg–N-CQD to the d-orbital of Hg(ii). Interestingly, the fluorescence of an aqueous solution of Mg–N-CQD containing Hg(ii) can be gradually restored in the presence of Cys due to the stronger binding affinity of Hg(ii) to Mg–N-CQD compared to Cys. The LOD for Hg(ii) was observed 0.02 μM, with a linear concentration range of 0.05 to 5 μM.^113^ A new type of nanosensor utilizing CH_3_NH_3_PbBr_3_ perovskite quantum dots (QDs) has been devised for detecting Hg(ii) ions. This sensor operates through a surface ion-exchange mechanism. The QDs produced emit vibrant green fluorescence when excited by 365 nm UV light. During interaction, Hg replaces a part of Pb on the surface of QDs, reducing the concentration of CH_3_NH_3_PbBr_3_ and leading to fluorescence quenching. Moreover, the fluorescence intensity of QDs remains unaffected by interfering metal ions, demonstrating the high selectivity and sensitivity of perovskite QDs for Hg(ii) detection. The LOD was calculated 0.124 nM within the concentration range of 0 nM to 100 nM.^114^

Recently, Jaiswal *et al.* developed starch-coated CuS quantum dots (QDs) for mercury detetction. The XRD technique was employed to verify the phase of CuS quantum dots (QDs), while UV-vis spectroscopy was utilized to characterize the LSPR peak in the near-IR region. TEM coupled with high-resolution analysis confirmed the formation of extremely small CuS QDs, ranging in size from 4 to 8 nanometers. CuS QDs exhibit selective and sensitive sensing of Hg(ii) ions *via* colorimetric changes. A significant selectivity for Hg(ii) ion detetction was observed over various metal ions, accompanied by a significant color change and a weakening of LSPR intensity.^115^ An comparative summary of quantum dot based nanosensor presented in Table 6.

**Table 6:** A summary of quantum dot-based nano-sensors for sensing of mercury metal ion

Entry	Nano sensor	Sensing approach	Linear range	LOD	Ref.
1	Zinc oxide quantum dots	Electrochemical	—	5 ppb	106
2	N, S-codoped graphene quantum dots	Fluorescent	0.1–15 μM	0.14 nM	107
3	Mn-doped ZnSe/ZnS quantum dots	Fluorescent	0 to 20 nM	0.1 nM	108
4	Quantum dot/DNA/gold nanoparticle	Fluorescent	—	1.2 ppb	109
5	MEA-functionalized GQDs (MEA-GQDs)	Fluorescent	0.05–5nM	10 nM	110
6	CdTe/CdS	Fluorescent	2 × 10^−9^ to 5 × 10^−7^ mol L^−1^	1.7 × 10^−9^ mol L^−1^	111
7	T–gCNQDs	Fluorescent	1.0 to 500 nM	0.15 nM	112
8	Mg–N-CQDs	Fluorescent	0.05–5 μM	0.02 μM	113
9	CH_3_NH_3_PbBr_3_ QDs	Fluorescent	0 nM to 100 nM	0.124 nM	114

### 2.6. Electrochemical nano-sensor for mercury

**Fig. 13:**
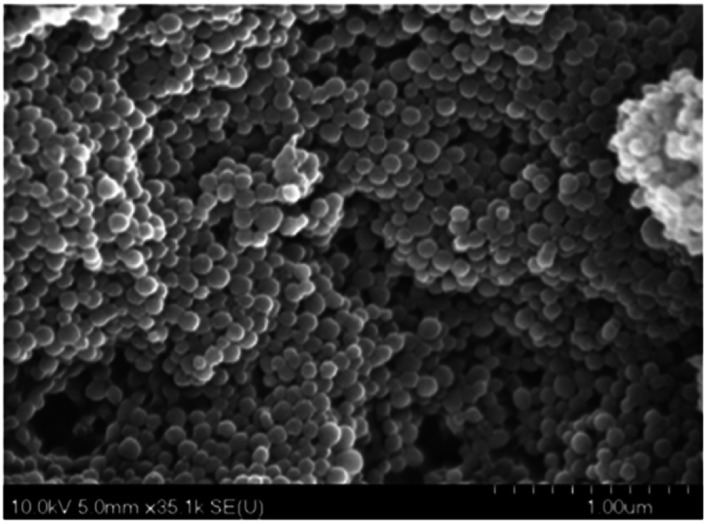
PANI nanospheres electrodeposited in a 1 mM aniline solution (pH = 5.0) are shown in a SEM picture (reproduced from ref. 117 with permission from Journal of Environmental and Toxicology, Copyright© 2017).

Electrochemical nano sensors designed for mercury sensing stand at the forefront of advanced detection methodologies, offering a reliable and sensitive approach. These sensors exploit the electrocatalytic properties of nanostructured materials, such as nanowires, nanotubes, or nanoparticles, to facilitate rapid and selective detection of mercury. Through precise control of the electrode surface and nanomaterial composition, these sensors can achieve improved sensitivity and specificity. The electrochemical response is triggered by the interaction between mercury ions and the modified electrode, resulting in measurable changes in current or potential. To detecting mercury(ii) in aqueous solution, Yanqin Yang *et al.* have devised an electrochemical biosensor based on a 3D-rGO@PANI nanocomposite. *In situ* chemical oxidative polymerization was used to create the 3D-rGO@PANI, which was subsequently used as the sensitive layer of a DNA adsorbent to detect Hg(ii) in aqueous solution. High affinity was shown by the amino-group-rich 3D-rGO@PANI for the immobilization of T-rich DNA strands, which bonded with Hg(ii) preferentially to form T–Hg(ii)–T coordination. With a LOD of 0.035 nM, the 3D-rGO@PANI nanocomposite demonstrated strong sensitivity and selectivity for Hg(ii) over a concentration range of 0.1 nM to 100 nM. Additionally, the nanocomposite exhibited selectivity and repeatability in the presence of other metal ions under conditions similar to those of Hg(ii) ions.^116^ Based on the creation of films of polyaniline nanoparticles, Etorki AM *et al.* have devised an electrochemical nanosensor for the detection of mercury ions from aqueous solutions. Polyaniline nanoparticles (PANI) were used to alter carbon electrodes that were screen-printed. FTIR, SEM, and XRD, were used to characterize the screen-printed carbon electrode modified by polyaniline nanoparticles in terms of both structure and morphology. As seen in Fig. 13, the PANI nanoparticles had a spherical shape and an apparent diameter that ranged from 20 to 45 nm. As determined, the LOD was 2.50 ± 0.03 ppb. The established LOD is less than the relevant WHO recommendation value.^117^

**Fig. 14:**
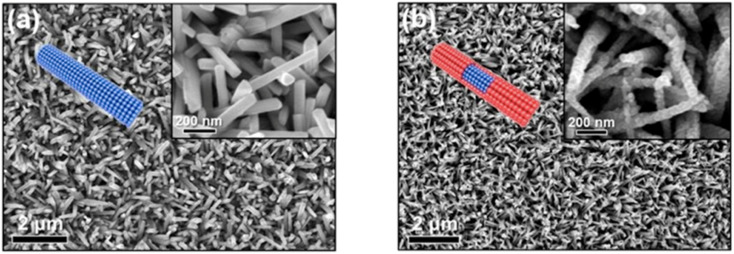
(a) SEM images of Te NWs before and (b) after the detection of 1 μM Hg(ii) ions (reproduced from ref. 118, with permission from Elsevier Ltd, Copyright © 2019).

A thermoelectric effect-based self-powered nanosensor for mercury ion detection has been disclosed by Y.-H. Tsao *et al.* Tellurium nanowires (TeNWs) serve as the sensing element's core material, spontaneously producing an electric output in response to a slight temperature differential in the environment. TeNWs also serve as a recognition element for reactions with mercury ions. With a linear concentration range of 10 nM to 1 μM, the developed thermoelectric nanosensor offers good sensitivity for the detection of Hg(ii) ions. Further SEM analysis, as illustrated in Fig. 14, provides changes in morphology while interacting with Hg(ii) ions. It has been observed that the surface morphology of Te NWs before and after the detection of 1 μM Hg(ii) ions changed from smooth to rough. In comparison to conventional sensors, the thermoelectric nanosensor exhibits significant potential to become a portable sensor for in-field detection of environmental samples due to its extra simplicity and low cost of production.^118^

Similarly, a unique self-powered triboelectric nanosensor has been developed by Snigdha Roy Barman *et al.* to detect Hg(ii) ions. Tellurium nanowire (Te NW) arrays are used in this solid–liquid contact electrification sensor as the sensing probe and the solid triboelectric material. Because of Te NWs' specific binding affinity for Hg(ii) ions, mercury telluride nanowires (HgTe NWs) spontaneously arise as a result of periodic contact and separation of Te NW arrays with the Hg(ii) solution. To improve the practicality of on-site sensing, Te NW arrays are attached to robotic hands that have an extra wireless transmission capacity. This allows for the quick identification of Hg(ii) ions in locations with limited resources using a simple “touch and sense” method. This innovative combination of robots and self-powered sensors has the prospect of developing automated chemical sensing devices at a reasonable cost for the on-the-spot identification of dangerous materials in the field.^119^ An comparative overview of electrochemical nanosensor presented in Table 7.

**Table 7:** An overview of electrochemical based nano-sensors for sensing of mercury metal ion

Entry	Nano sensor	Sensing approach	Linear range	LOD	Ref.
1	3D-rGO@PANI	Electrochemical impedance	0.1 nM to 100 nM	Of 0.035 nM	116
2	PANI nanoparticles	Electrochemical *via* square wave anodic stripping voltammetry	—	2.50 ± 0.03 ppb	117
3	Te NWs	Thermoelectric	10 nM to 1 μM	1.7 nM	118

### 2.7. Silica based nano-sensor for mercury

**Scheme 11:**
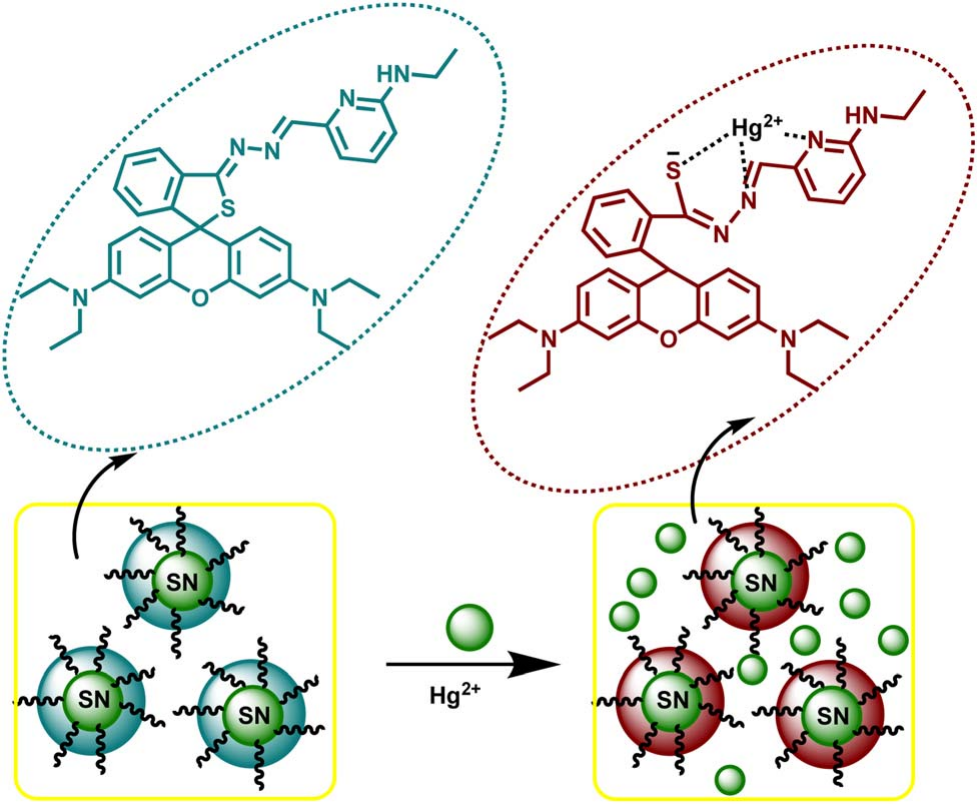
Schematic illustration of Hg(ii) binding with rhodamine-functionalized silica nanoparticles (RFSNP).

Silica's inherent biocompatibility, stability, and its versatility make it an ideal candidate for designing nano sensors with enhanced performance. Further, due to small size, high surface area-to-volume ratio, and tunable surface chemistry, silica-based nano sensors offer a favorable possibility for the sensitive and selective detection of mercury ions, bringing forth a robust platform that combines the advantageous properties of silica NPs. Through surface modification with specific ligands or receptors, these sensors gain the ability to selectively interact with mercury ions, achieving enhanced sensitivity. Such as, rhodamine-functionalized SiNPs (RFSiNP) designed for sensing Hg(ii), H_2_PO_4_^−^, and S^2−^*via* different combination mechanisms. The rhodamine unit in the sensor functions as a donor of hydrogen bonds for sensing as well as a metal binding site. The emission spectrum response in the presence and absence of excess metal ions served as confirmation that RFSiNP bound to metal ions. RFSNP exhibited a modest emission band at 565 nm, with a quantum yield of around 0.04 for the rhodamine (spirocyclic form). When Hg(ii) was added, it was increased by almost thirteen times with a redshift of 13 nm to 578 nm. The emission color changed to red (spirocyclic ring opening) and was visible to the unassisted eye. Interestingly, Scheme 11 shows that the ring-opening of the rhodamine unit was caused by Hg(ii) binding. High selectivity for Hg(ii) ions was demonstrated by RFSiNP over competing metal ions.^120^

J. Isaad and A. El Achari *et al.* devised a hybrid substance tailored for Hg(ii) detection, which integrates a derivative of the dansyl fluorophore along with an aza-thia crown ether segment attached to silica nanoparticles (SiNPs). Upon binding, this nanosensor displayed robust Hg(ii) selective “on–off” fluorescence switching. The detection limit for Hg(ii) in a mixture of ethanol and water at neutral pH was determined to be 10^−7^ M. Importantly, other metal ions presence did not affect the nanosensor's selectivity.^121^

**Scheme 12:**
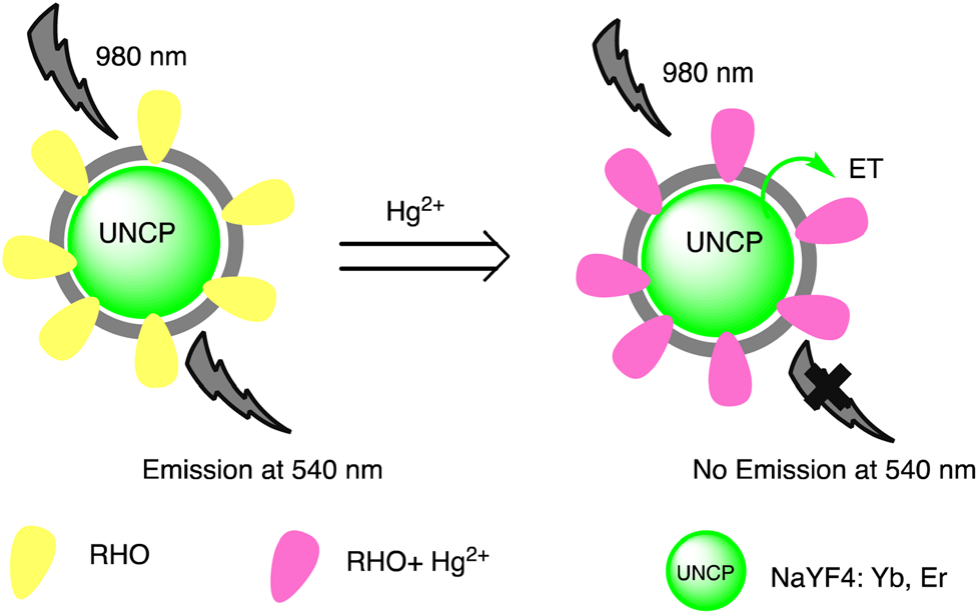
Graphic for luminescence resonance energy transfer between NaYF4:Yb, Er UCNPs and the probe RHO for Hg(ii) ion sensing.

Similarly, a dual-emission fluorescence probe based on silica, labeled as Tb-DPA@SiO_2_–Eu-DPA, has been employed for the detection of Hg(ii) ions. To evaluate the sensing sensitivity of probe, the probe's fluorescence responses to different concentrations of Hg(ii) were investigated. The fluorescence intensity of the probe at 615 nm showed particular sensitivity to Hg(ii), decreasing with increasing concentrations. However, the fluorescence intensity at 545 nm remained nearly constant. The intensity ratio of *F*_545_/*F*_615_ exhibited a strong linear correlation with Hg(ii) concentrations reaching from 10 nM to 2 μM. The LOD was determined to be 7.07 nM, which falls below the maximum level (10 nM) of Hg(ii) in drinking water approved by the US EPA.^122^ L. Feng *et al.* produced a mesoporous SiNP-encapsulated conjugated polymer poly(phenylene vinylene) (PPV) and a spirolactam rhodamine-B derivative to create a FRET-based ratiometric sensor for mercury ions called PPV@MSN@SRhB. In this setup, PPV acts as the donor. This nanosensor has been found to be highly sensitive to Hg(ii) ions within pH values ranging from 5 to 8, with a LOD of approximately 200 nM. The detection of Hg(ii) was achieved by monitoring the fluorescence emission spectra of PPV@MSN@SRhB particles following the introduction of varying quantities of Hg(ii). With the introduction of Hg(ii) ions, there was a gradual decline in the emission of PPV within the 502–536 nm range, accompanied by the emergence of new fluorescence emission from rhodamine B (in its open-ring state) at 585 nm. In this procedure, the transformation occurs where the rhodamine group of spirolactam transitions into the ring-opened state as a result of interaction with Hg(ii) ions. Consequently, the fluorescence of the PPV donor diminishes as it transmits its energy to the ring-opened RhB *via* FRET.^123^ Similarly, a rhodamine and silica-based sensor titled UCNPs@SiO_2_-RHO has been developed by L. Wang, T. Wang, M. Shao *et al.* for mercury sensing. The preparation involved the modification of rhodamine hydrazide with glyoxal and covalently grafting it onto the SiO_2_ layer of UCNPs@SiO_2_, as illustrated in Scheme 12. When the UCNPs@SiO_2_-RHO nanocomposites were exposed to infrared light (980 nm), they exhibited a significantly high green up-conversion luminescence visible to the naked eye. Additionally, the sensing ability of sensor for Hg(ii) was investigated by introducing various concentrations of Hg(ii) ions under 980 nm laser excitation. With the increasing concentration of Hg(ii) ions (ranging from 0 to 35 μM), there was a pronounced decrease in the green emission intensity of the particles. This decrease signifies an energy transfer route from up-conversion nanoparticles (UCNP) to rhodamine (RHO), which is bound to Hg(ii). Notably, the LOD for this phenomenon was determined to be 2.4 μM.^124^

**Scheme 13:**
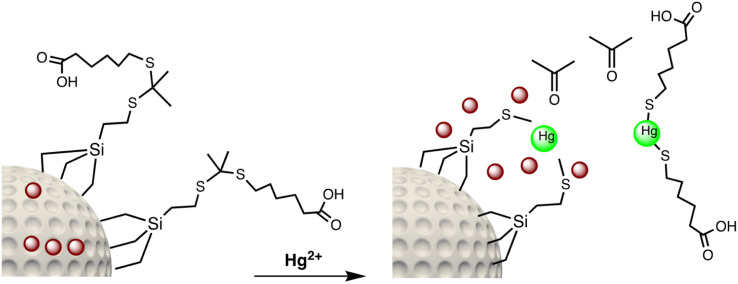
Graphic for Hg(ii) ions detection mechanism.

Moreover, a pH-responsive micro-object optical sensor for Hg(ii) detection was utilized, employing a framework of 3D gyroidal mesoporous aluminosilica. This sensor incorporated a dressing receptor modified with a porphyrinic moiety for enhanced functionality. The colorimetric approach was utilized to detect and remove metal ions from the aluminosilica nanosensor, and the results were validated using ICP-MS. The optical sensor pellets made it possible to quickly (less than 60 seconds) detect small concentrations of Hg(ii), Cd(ii), and Cu(ii) ions (10^−11^ mol dm^−3^). This detection was based on a colorimetric signal that was observable using UV-vis reflectance spectroscopy and was visible to the naked eye.^7^ S. Jimenez-Falcao *et al.* developed a nanosensor based on mesoporous silica nanoparticles (MSN) to enable rapid and straightforward colorimetric assessment of inorganic mercury ions. The nanodevice was constructed by initially loading MSN with tris(2,20-bipyridyl)dichlororuthenium(ii) (Ru(bipy)2) as a reporter dye, followed by modification using (3-mercaptopropyl)trimethoxysilane. Subsequently, the resulting nanomaterial was coated with a novel gate-like molecular ensemble sensitive to Hg(ii) based on a dithiocetal derivative. The nanosensor can detect Hg(ii) in concentrations ranging from 154 pM to 31 nM using a process shown in Scheme 13. When Hg(ii) ions contact with the solid sensor, they break the linear dithioacetal linkages, resulting in the production of new Hg(S–R)_2_ derivatives. This has a LOD of 60 pM and a sensitivity of 29.9 a.u per μM.^125^

**Scheme 14:**
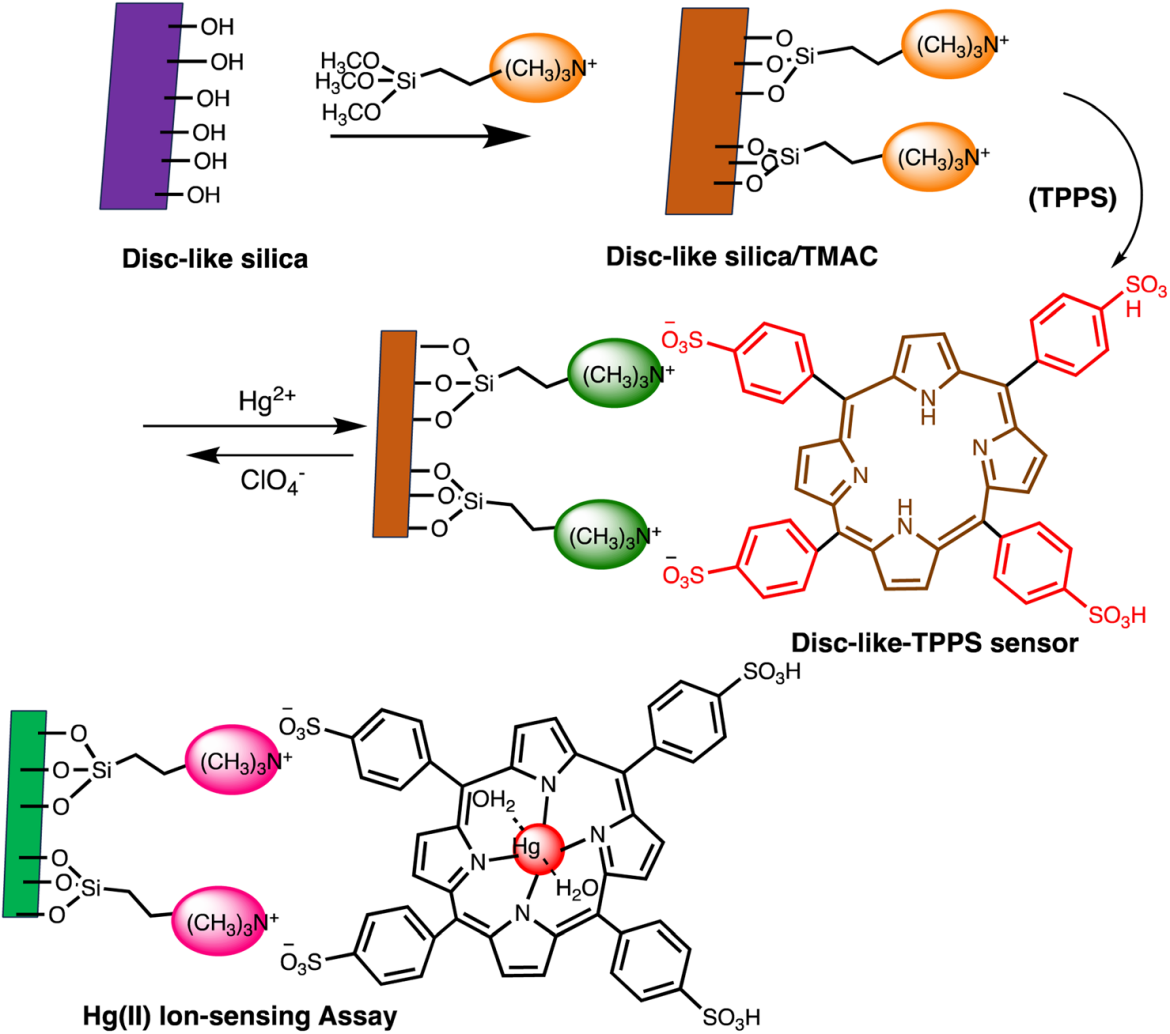
Schematic representation of disc-like-TPPS sensor preparation and Hg(ii) sensing.

Recently, a novel organic–inorganic hybrid nanosensor, named MSN@Py-EOA, incorporating pyrene-functionalization, has been presented to detect Hg(ii). Utilizing mesoporous silica nanoparticles as the framework and pyrene units as the signaling component, this hybrid nanosensor demonstrates a distinct “turn-off” reaction selectively towards Hg(ii) ions in contrast to a range of other metal ions. The nanosensor is capable of detecting Hg(ii) at concentrations ranging from 1 to 20 μM, with a detection limit of 0.62 μM.^126^ Similarly, a fluorescent mesoporous silica hybrid nanosensor system utilizing ethidium bromide (MSH@EB NPs) was developed for the selective detection of Hg(ii) ions by Santhamoorthy Madhappan, *et al.*^127^ When exposed to Hg(ii) ions, the sensor's optical color changes from pink to green. The produced MSH@EB NPs system may be utilized to selectively monitor harmful Hg(ii) ions in aqueous solutions and biological samples, with a LOD of 100 ppb in a short period of time. Furthermore, El-Safty *et al.* developed nano-sized monoliths (disc-like) as optical sensing platform for Hg(ii) ions detection. By immobilizing the TPPS probe and applying grafted-controlled surface modification of cage-like monoliths with *N*-trimethoxysilylpropyl-*N*,*N*,*N*-trimethylammonium chloride (TMAC), optical sensors are fabricated in two steps. This organic–inorganic monolithic disc enhances procedures significantly, enabling enhanced levels of instrument control and data processing. It enhances sensitivity, offers versatility, and enhances selectivity for Hg(ii) ion binding tests. Integrating the TPPS chromophore into the disc-shaped monolithic HOM-9 mesopore structures led to the development of optical chemical nanosensors, as depicted in Scheme 14. These nanosensors exhibit notable efficiency in sensing Hg(ii) ions, demonstrating sensitivity, selectivity, and rapid response times. Using disc-like sensors, the quantification and detection limits of Hg(ii) ions were determined to be 4.0 ppb and 1.2 ppb, respectively.^128^ An comparative overview of silica-based nanosensor presented in Table 8.

**Table 8:** An overview of silica-based nano-sensors for sensing of mercury metal ion

Entry	Nano sensor	Sensing approach	Linear range	LOD	Ref.
1	RFSNP	Rhodamine ring-opening reaction	—	—	120
2	Aza-thia crown ether grafted silica nanoparticles (SiNPs)	On–off type fluorescence switching		10^−7^ M	121
3	Tb-DPA@SiO_2_–Eu-DPA	Fluorescence emission	10–2 μM	7.07 nM	122
4	PPV@MSN@SRhB	Fluorescence emission	—	200 nM	123
5	UCNPs@SiO_2_–RHO	Luminescence	—	2.4 μM	124
6	MSN-based nano-sensor	Hg^2+^ ions disrupt the linear dithioacetal linkages upon interaction	154 pM to 31 nM	60 pM	125
7	MSN@Py-EOA	Turn-off fluorescence	1 to 20 μM	0.62 μM	126
8	MSH@EB NPs	Optical and fluorescence	—	100 ppb	127
9	Disc-TMAC–TPPS sensor	Optical sensing	—	1.2 ppb	128

### 2.8. Magnetic nano-sensor for mercury sensing

Owing to unique magnetic properties, small size, and surface functionalities, magnetic nanoparticles (MNPs) are widely used for mercury sensing. Further, magnetism enables easy separation and retrieval of the sensing platform from complex sample matrices. Number of magnetic nano sensor have been explored by researchers in past for mercury sensing. For instance, using two different sizes of MNPs, Yunyun Hu *et al.* have developed an assay for Hg(ii) using a Magnetic Resonance Spectroscopy (MRS) platform assisted by magnetic separation. MB30-DNA1 is used as the MRS probe and MB200-DNA2 is used as the magnetic capture probe to precisely measure Hg(ii). This tactic relies on the precise and potent interaction of mercury ions with the T–T mismatch seen in duplexes of double-stranded DNA. This contact is facilitated by oligonucleotide functionalized MNPs, which operate as both the magnetic capture probe and the MRS signal probe. The results show that Hg(ii) ions may be successfully detected in tap water, lake water, and serum by using the concentration-dependent MRS sensing mechanism of MNPs. This can increase the detection range and improve accuracy. The LOD was calculated 0.23 μM, with a linear concentration range of Hg(ii) from 0.8 μM to 50 μM.^129^

**Scheme 15:**
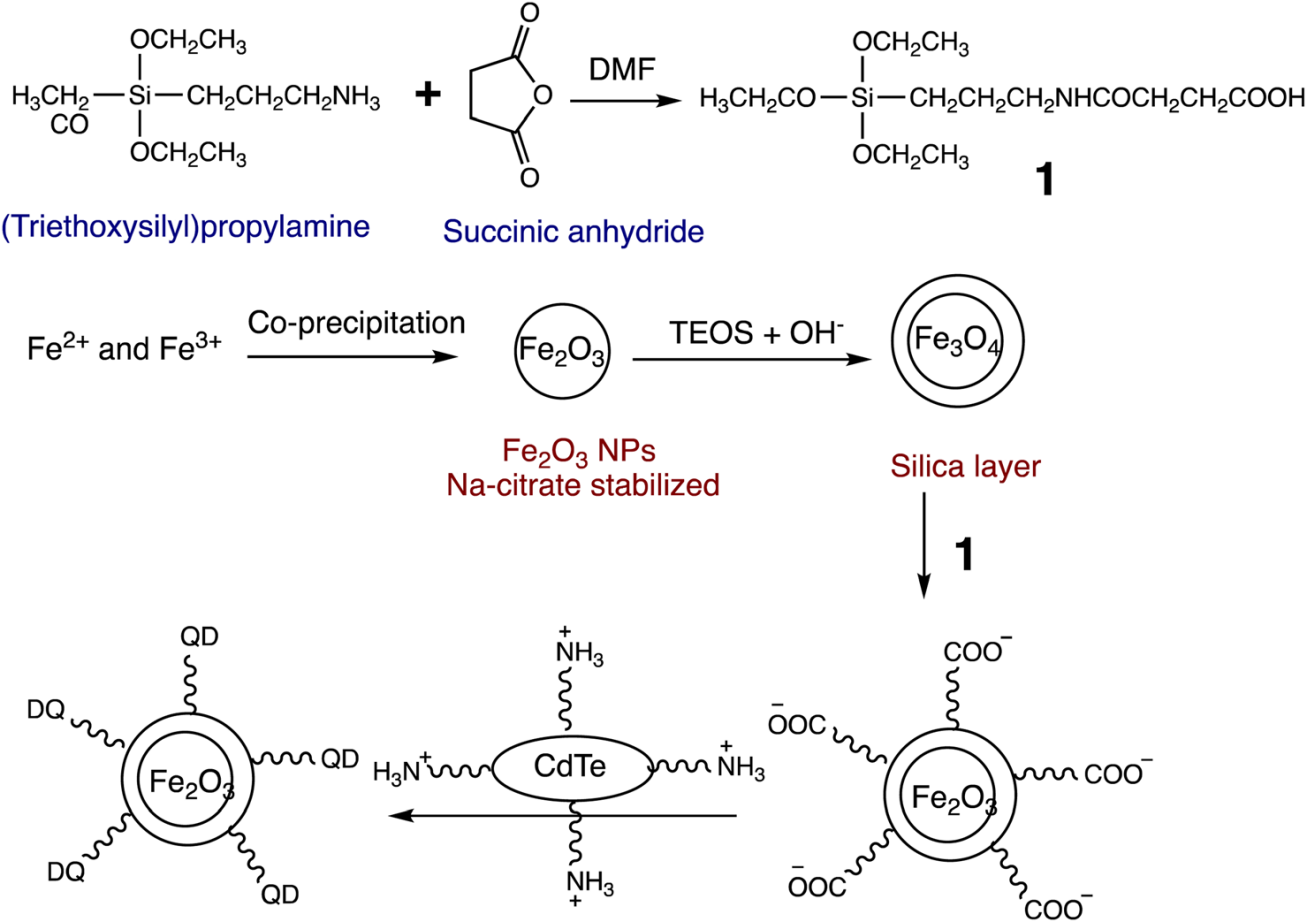
Graphic for multimodal nanosensor preparation.

A fluorescence sensing nanosensor for Hg(ii) detection has been devised by Wei Wang and co-workers. It uses adamantane-modified inclusion complex magnetic nanoparticles (TFIC MNPs) and TSRh6G-*b*-cyclodextrin. When these sensors come into contact with Hg(ii) metal ions, they display distinct ‘turn-on’ fluorescence improvements as well as a visible color change from light brown to pink. Even at micromolar concentrations in aqueous solutions, TFIC MNPs exhibit exceptional selectivity and sensitivity in the detection of Hg(ii) ions due to their large surface area and superior permeability. Specifically, the detection limit is measured at 5.04 × 10^−6^ mol L^−1^. Even though this method goes beyond the EPA's criteria for mercury in drinking water, it is still a viable approach for the detection of high concentration Hg(ii) ions.^130^ Furthermore, a research group has explored multimodal nanosensors for the efficient detection and removal of Hg(ii) ions in aqueous media. This nanosensor, fabricated through a multistep synthesis process outlined in Scheme 15, consists of a thin silica shell encompassing magnetic (Fe_2_O_3_) nanoparticles, a stationary spacer arm, and fluorescent quantum dots. This facilitates the concurrent detection and subsequent removal of identified mercury. The surface of the initial nanosensor model is altered with (triethoxysilyl propyl–carbamoyl) butyric acid to impart a negative charge. Positively charged cadmium telluride (CdTe) quantum dots capped with cysteamine are then electrostatically attached to the surface. The multimodal nanosensor that was produced is used to identify Hg(ii) ions in an aqueous medium. The nanosensor's photoluminescence emission intensity decreases with increasing Hg(ii) concentration, leading to a notable reduction in signal strength. Because of their great affinity for nitrogen atoms, Hg(ii) ions adsorb onto the surface of quantum dots *via* cysteamine's functional amide groups, which facilitates the transfer of electrons from quantum dots to Hg(ii). These nanosensors are capable of detecting Hg(ii) ions at the nanomolar level with a LOD of 1 nm.^131^

**Scheme 16:**
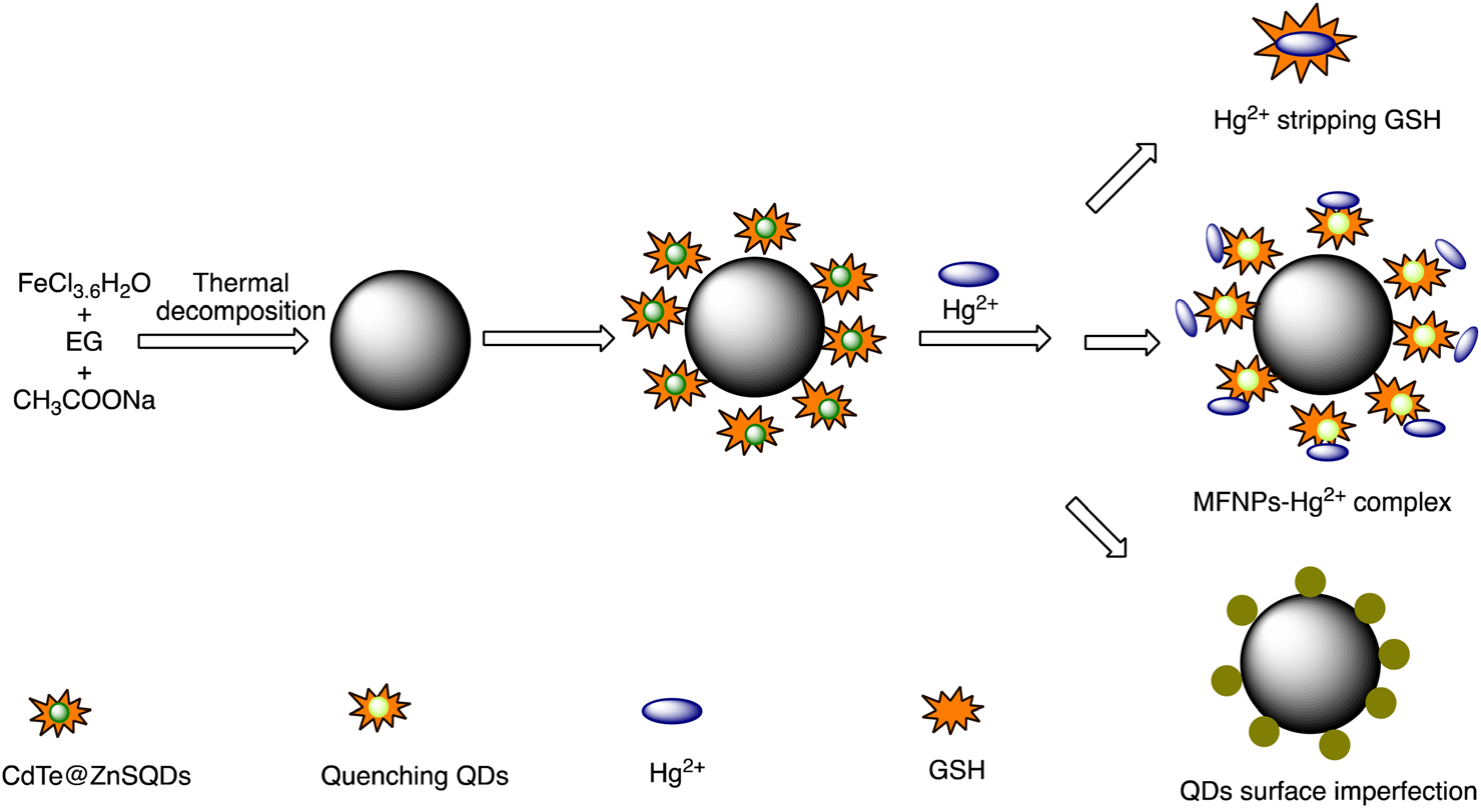
Schematic illustration for interaction of MFNPs with Hg(ii).

Moorthy Suresh *et al.* have established a highly ordered rhodamine-functionalized magnetic nanomaterial for mercury detection. This material exhibits finely tuned three-dimensional cage-like mesopores and ferrosilicate frameworks with ultrafine Fe_2_O_3_ particles dispersed uniformly across the mesoporous channels. The FeKIT-5 materials' silanol groups that terminate the surface are chemically bonded to the highly luminescent rhodamine receptor (3′,6′-bis(ethyl-amino)-2′,7′-dimethyl-2-((thiophen-2-ylmethylene)amino)spiro[isoindoline-1,9′-xanthen]-3-one), which facilitates the detection of Hg(ii) in aqueous environments. Fluorescence spectra changes, depending on the amount of Hg(ii) ions present, show a slow increase in fluorescence intensity at 560 nm when excited at 500 nm. This increase is explained by the conversion of the highly fluorescent xanthene form from the non-fluorescent spirolactam form. The LOD was calculated 0.1 ppm.^132^ Similarly, Yang *et al.* developed magnetic-fluorescent nanocomposites functionalized with carboxymethyl chitosan to detect and remove Hg(ii) ions at the same time. When Hg(ii) ions are present, the nanosensor's fluorescence may be significantly suppressed. Under ideal conditions, it was shown that the quenching effect of Hg(ii) on the fluorescence of the nanosensor depended on concentration, ranging from 3 × 10^−7^ to 5 × 10^−6^ mol L^−1^. The detection limit of Hg(ii) was determined to be 9.1 × 10^−8^ mol L^−1^. During the detection process, Hg(ii) was introduced into the MFNPs system, leading to effective quenching of the fluorescence of the obtained nanocomposite sensor. Scheme 16 illustrates the three different mechanisms that contribute to the quenching effects: the production of non-fluorescent ground-state MFNPs-Hg(ii) complexes, the competitive removal of surface ligands GSH by Hg(ii), and surface defects on the quantum dots (QDs). Additionally, magnetic-fluorescent nanocomposite sensors have the capability to recover the metal ions through attraction from external magnetic fields.^133^

**Fig. 15:**
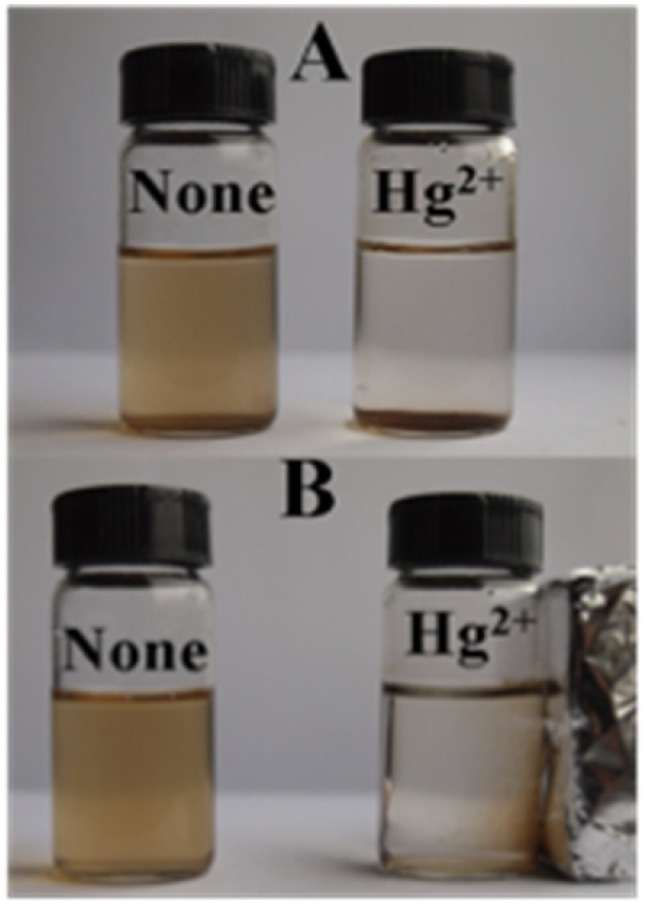
Separation of Hg^2+^-bound nano sensor from aqueous *via* using a magnet (A) before apply a magnet and (B) after apply a magnet for separation (reproduced from ref. 134 with permission from Elsevier Inc, Copyright © 2012).

Jie Zhao *et al.* have devised a magnetic fluorescent sensor functionalized with 1,8-naphthalimide for the purpose of detecting and eliminating Hg(ii) within aqueous solutions. Notably, this nanosensor demonstrated significant fluorescence quenching capabilities along with a pronounced selectivity for Hg(ii) ions in comparison to other metal ions present in water. Conversely, it has observed that Fe_3_O_4_-based fluorescence sensor function as a suitable adsorbent for the removal of Hg(ii) through nanoparticle aggregation or magnetic separation. The LOD was found 1.03 × 10^−8^ M. Additionally, the Fe_3_O_4_-based fluorescent sensor showed ease of aggregation and complete self-settlement after half an hour, as depicted in Fig. 15. Utilizing a magnet, the Hg(ii)-bound nanosensor could be effortlessly separated from the aqueous solution.^134^

**Scheme 17:**
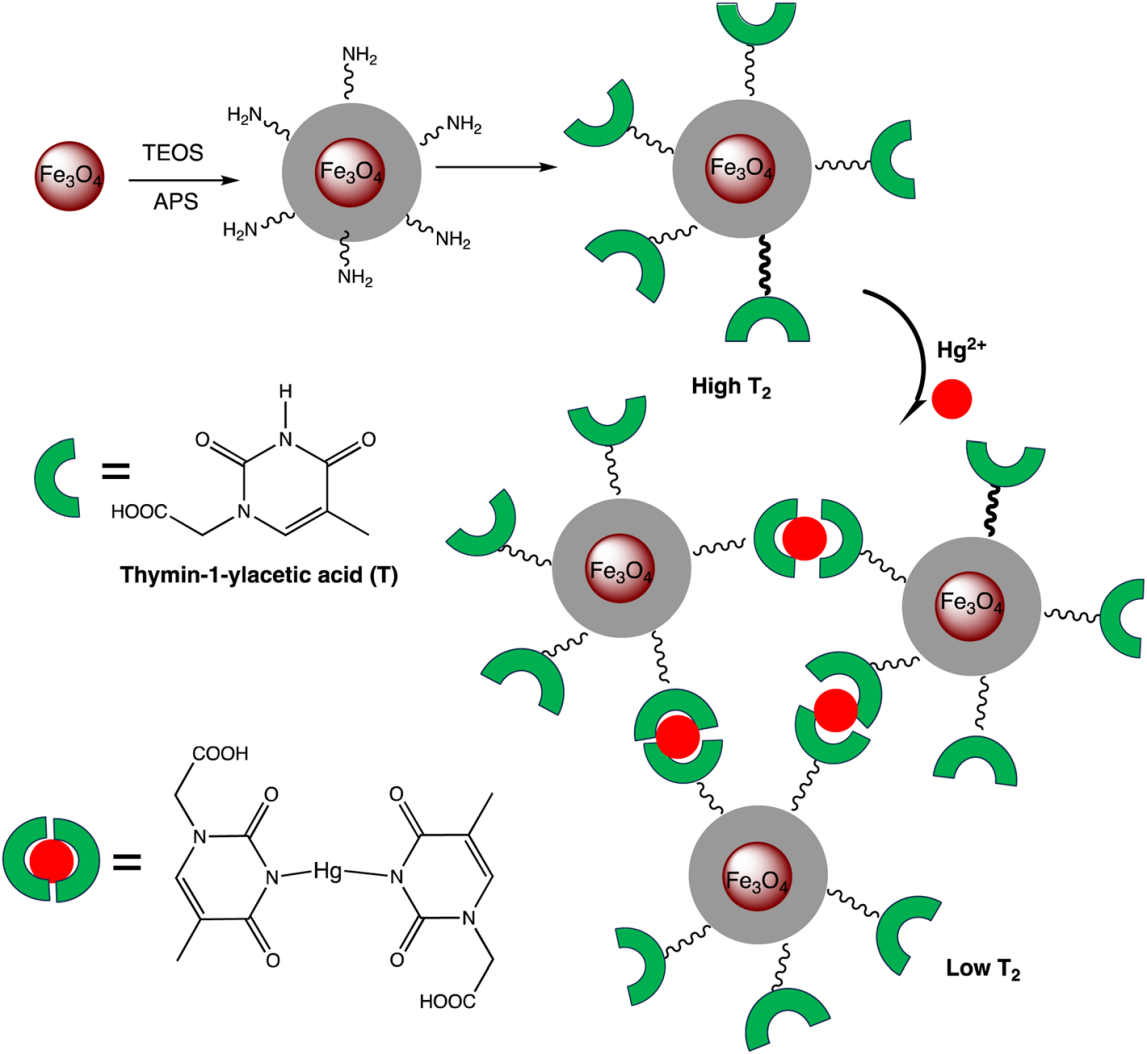
Graphic for Fe_3_O_4_@SiO_2_–T NPs preparation and Hg(ii) detection mechanism.

**Scheme 18:**
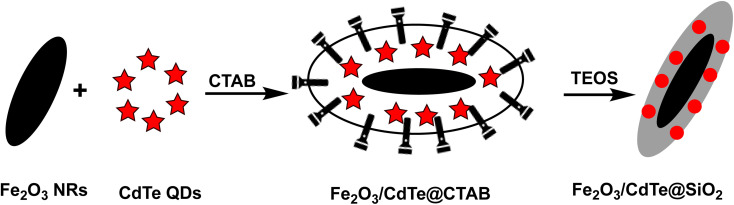
Graphic for CdTe/Fe_2_O_3_@SiO_2_NPS preparation.

Thymidine-functionalized supermagnetic iron oxide nanoparticles (Fe_3_O_4_@SiO_2_–TNPs) have been developed by Hong Yang *et al.*, as illustrated in Scheme 17, for mercury sensing. The detection method is related to the Hg(ii)-induced nanoassembly of Fe_3_O_4_@SiO_2_–TNPs, which results in a decrease in the transverse relaxation time (*T*_2_) of nearby water protons. When Hg(ii) ions added to a solution of Fe_3_O_4_@SiO_2_–TNPs, a noticeable decrease in *T*_2_ was observed. The changes in *T*_2_ are highly sensitive to the concentration of Hg(ii). The diameter of Fe_3_O_4_@SiO_2_–TNPs was measured using dynamic light scattering (DLS), revealing an average dispersed NP diameter of approximately 114 nm. When exposed to 50 μM Hg(ii), the mean diameter of nanoparticles promptly increased to about 213 nm, accompanied by minimal quantities of larger nanoparticles. This alteration in nanoparticle size arises from the nanoassembly prompted by thymine–Hg–thymine interactions, as depicted in Scheme 17. These findings robustly imply that Hg(ii) has the capacity to instigate the nanoassembly of Fe_3_O_4_@SiO_2_–TNPs.^135^ Wang, Li *et al.* produced core/shell nanostructures consisting of CdTe/Fe_2_O_3_@SiO_2_ for the purpose of magnetically assisted photoluminescent sensing of Hg(ii) ions. The fabrication procedure entails the synthesis of magnetic Fe_2_O_3_ nanorods (NRs) and CdTe quantum dots (QDs) coated with l-cysteine (l-Cys), subsequently encapsulated within SiO_2_, as illustrated in Scheme 18. The mesoporous SiO_2_ shell formed in the nanocomposites not only encloses the CdTe quantum dots but also allows entry for small molecules and ions to interact with the quantum dots, triggering a fluorescence reaction. The core/shell nanostructures of CdTe/Fe_2_O_3_@SiO_2_ demonstrate a rod-like, one-dimensional structure with an average diameter of around 100 nm and lengths spanning from 500 to 700 nm. As the concentration of Hg(ii) ions increases, the fluorescence emitted by CdTe QDs decreases significantly. The reduction in fluorescence intensity of Hg(ii) ions is attributed to a combination of static and dynamic quenching mechanisms. The core/shell nanocomposites demonstrate high sensitivity compared to conventional methods for Hg(ii) ions within the concentration range of 0.1 to 10 mM.^136^

**Scheme 19:**
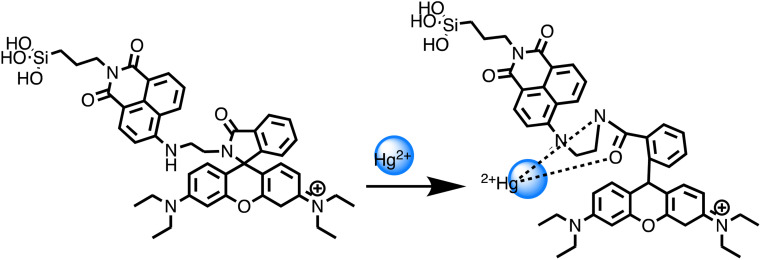
Mechanism of interaction of chromogenic NR with Hg^2+^.

A novel “turn-on” nanosensor, CoFe_2_O_4_@SiO_2_ developed for Hg(ii) sensing by M. Gharagozlou and S. Rouhani. The synthesis of the nanosensor entails attaching 1,8-naphthalimide dye conjugated with rhodamine dye onto cobalt nano ferrite particles. The resulting CoFe_2_O_4_@SiO_2_/NR nanocomposite displayed specific ‘turn-on’ fluorescence enhancements upon exposure to Hg(ii) ions. The addition of Hg(ii) caused a sharp decrease in the fluorescence intensity at 480 nm as well as the appearance of a new red-shifted emission band at about 580 nm, which was attributed to the rhodamine acceptor's emission. This energy transfer was accompanied by a resonant color shift from green to red when illuminated at 360 nm. The intensity of fluorescence emission at 580 nm increased and at 480 nm decreased respectively. Furthermore, it was observed that the increase in fluorescence at 580 nm is commonly linked to the opening of the spirolactam ring of rhodamine-6G as illustrated in Scheme 19. A linear range of probe response was obtained between 0.04–0.76 μM of Hg(ii) ions, indicating its potential use in real tap samples.^137^

Niu, He, and co-workers investigated the peroxidase mimicking activity triggered by Hg(ii) ions in cysteine–decorated ferromagnetic particles (Cys–Fe_3_O_4_) for mercury detection. The Cys–Fe_3_O_4_ prepared exhibited minimal peroxidase-like activity, attributed to the coordinated interaction between Cys and Fe, as well as the obstruction of active sites on Fe_3_O_4_ by the external layer of Cys. However, in the presence of Hg(ii) ions, the Cys bound to the surface of the Fe_3_O_4_ particles may be displaced due to a stronger coordination between Cys and Hg(ii), thereby uncovering active Fe_3_O_4_ sites capable of catalyzing the oxidation of colorless 3,3′,5,5′-tetramethylbenzidine (TMB) to yield blue TMBox. Later, the Cys–Fe_3_O_4_ particles were utilized to fabricate a colorimetric Hg(ii) nanosensor. It has been noticed, the produced enzyme-mimicking biosensor enabled the detection of Hg(ii) ions within a linear span ranging from 0.02 to 90 nM, achieving a LOD of 5.9 pM. This LOD is achieved in this study is notably below the WHO's permissible maximum levels of mercury in drinking water, which is 5 nM.^138^ Recently, Süreyya Oğuz Tümay *et al.* reported fluorescent iron oxide nanoparticles (Py@Fe_2_O_3_) for mercury sensing. By using a click reaction to modify the surface of Fe_2_O_3_ with pyrene-based fluoroionophore groups, the sensor was created. The sensor and fluoroionophore were characterized morphologically, structurally, and thermally using various spectroscopic techniques. The nanosensor exhibited a “turn-off” fluorescence response to Hg(ii) with a linear range of 0.010–1.000 μmol L^−1^ and a LOD of 3.650 nmol L^−1^. The measured LOD was found to be below the allowable threshold for Hg(ii) in drinking water established by the WHO.^139^

**Scheme 20:**
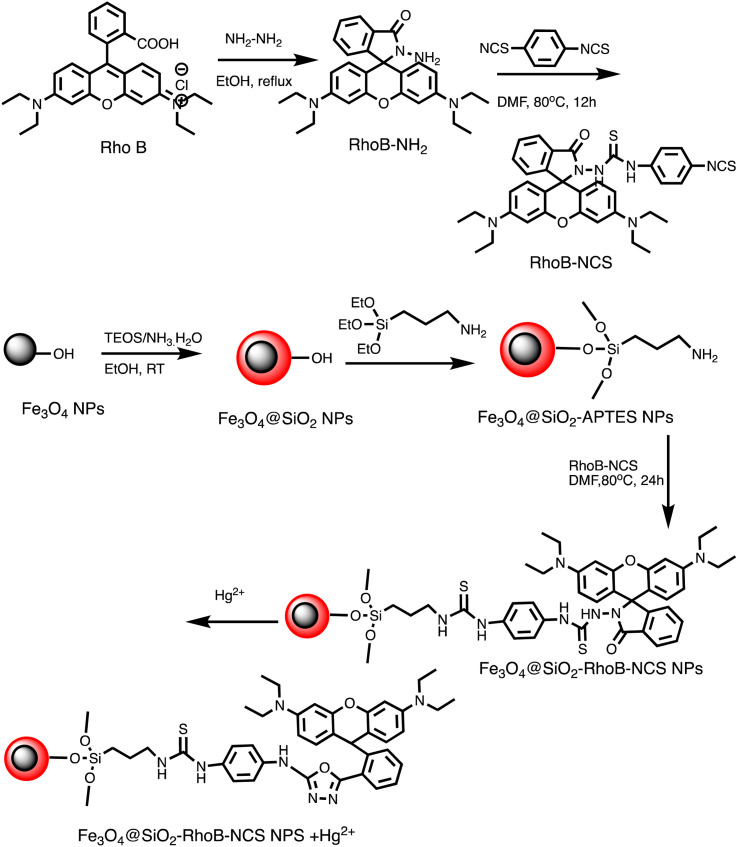
Schematic illustration for Fe_3_O_4_@SiO_2_-RhoB-NCS preparation and Hg(ii) sensing.

**Scheme 21:**
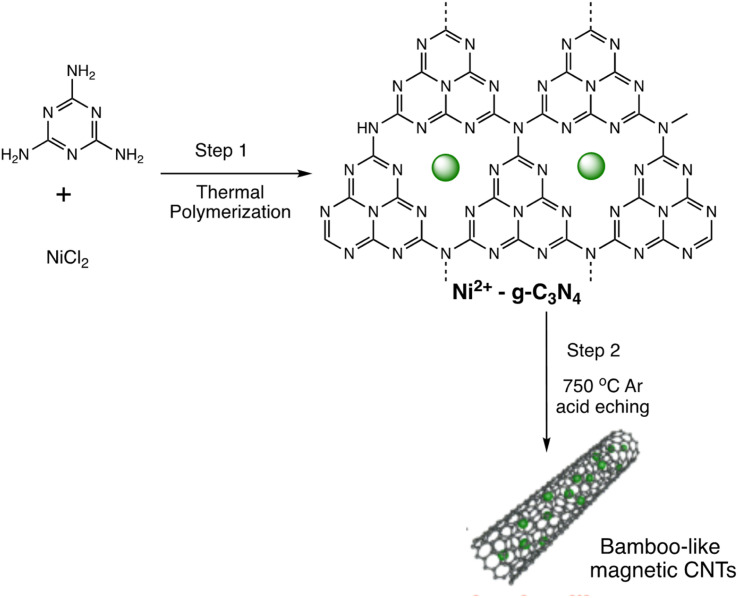
Synthetic procedure for BMCNTs preparation.

In another study, a rhodamine derivative (RhoB-NCS) was developed by Xianfei Zeng *et al.* and immobilized on the surface of Fe_3_O_4_@SiO_2_ nanoparticles (NPs) to investigate mercury sensing. The synthetic route is depicted in Scheme 20. Fe_3_O_4_@SiO_2_NPs served as a fluorescent sensor for the detection and removal of Hg(ii) ions. A noticeable change in color from orange to reddish-brown was noticed without the aid of magnification (naked eyes), with a detection threshold as low as 1.0 × 10^−6^ M.^140^ Shunru Jin *et al.* developed an innovative fluorescent biosensor employing bamboo-like magnetic carbon nanotubes (BMCNTs) in conjunction with FAM-labeled T-rich single-stranded DNA (ssDNA) for the effective detection of Hg(ii) ions in aqueous solutions. The synthesis of BMCNTs involved two major steps, as depicted in Scheme 21. The first step involved the formation of a Ni(ii)–grafted graphitic C_3_N_4_ composite *via* thermal polymerization, followed by the second step of tubular structure formation *via* acid etching of Ni(ii)–g-C_3_N_4_ at 750 °C. TEM image analysis clearly confirmed the bamboo-like morphology of the carbon nanotubes (CNTs). The biosensor effectively detects Hg(ii) in the linear range of 0.05–1 μM with a LOD of 20 nM. FAM-labeled ssDNA adsorbs on BMCNTs through H-bonding and π–π stacking, resulting in fluorescence quenching. In the presence of Hg(ii), the formation of double-stranded dsDNA through the T–Hg(ii)–T interaction enables the removal of FAM-labeled ssDNA from the surface of BMCNTs, leading to fluorescence recovery. In addition, the biosensor shows the ability to separate Hg(ii) ions with minimal response to other metal ions. Interestingly, BMCNTs can be easily separated and reutilized using an external magnet, providing a more economical, user-friendly, and sustainable method for detecting Hg(ii) ions.^141^ An comparative overview of magnetic nanosensor presented in Table 9.

**Table 9:** An overview magnetic nano-sensors for sensing of mercury metal ion

Entry	Nano-sensor	Sensing approach	Linear range	LOD	Ref.
1	Magnetic-separation-assisted MRS platform	MRS sensing	0.8 μM and 50 μM	0.23 μM	129
2	TFIC MNPs	Fluorescent	—	5.04 × 10^−6^ mol L^−1^	130
3	Multimodal MNPs nanosensor	Fluorescent	—	1 nm	131
4	Magnetic mesoporous ferrosilicate nanocages functionalized with rhodamine	Fluorescent	—	0.1 ppm	132
5	Carboxymethyl chitosan-Functionalized magnetic-fluorescent nanocomposites	Fluorescent	3 × 10^−7^ to 5 × 10^−6^ mol L^−1^	9.1 × 10^−8^ mol L^−1^	133
6	1,8-Naphthalimide-@MNPs	Fluorescent	—	1.03 × 10^−8^ M	134
7	CdTe/Fe_2_O_3_@SiO_2_ core/shell nanostructures	Fluorescent	01 to 10 mM	—	136
8	CoFe_2_O_4_@SiO_2_	Fluorescent	0.04–0.76 μM		137
9	Cys-Fe_3_O_4_ MNPs	Fluorescent	0.02–90 nM	5.9 pM	138
10	Py@Fe_2_O_3_	Fluorescent	0.010–1.000 μmol L^−1^	3.650 nmol	139
11	Fe_3_O_4_@SiO_2_-RhoB-NCS	Fluorescence	—	1.0 × 10^−6^ M	140
12	BMCNTs	Fluorescence quenching	0.05–1 μM	20 nM	141

### 2.9. Organic scaffold derived nano-sensor for mercury

**Scheme 22:**
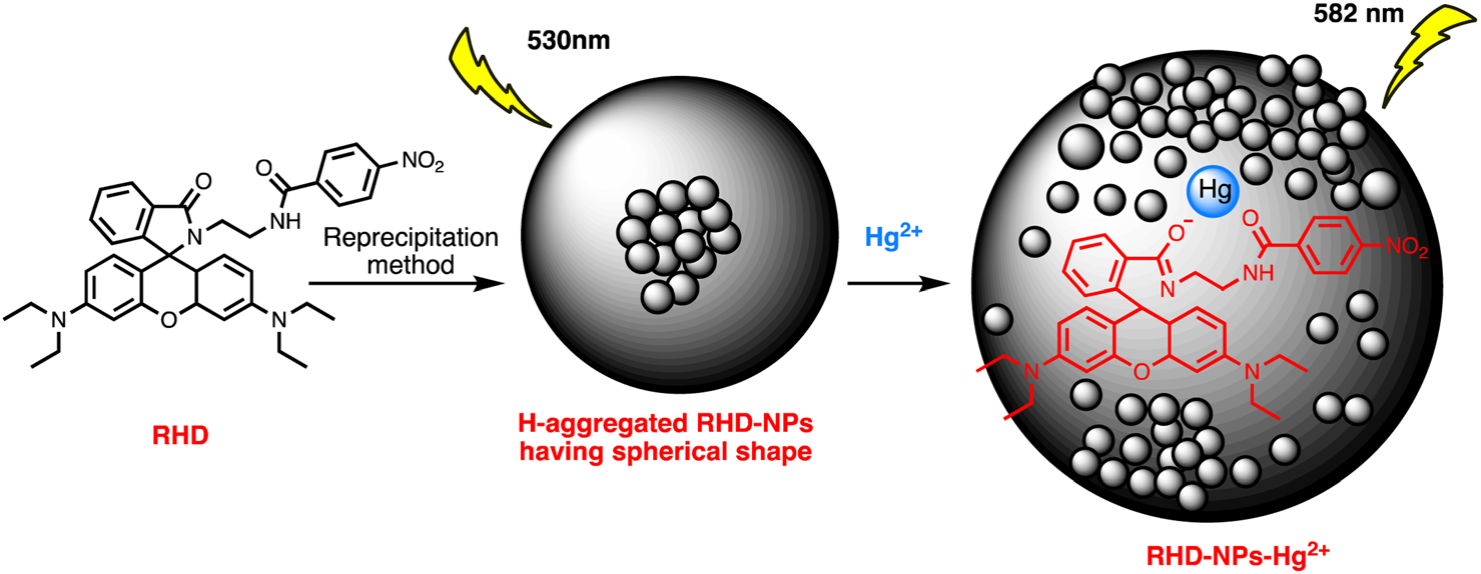
Formation of RHD-Ps and RHD-NPs-Hg^2+^ complex.

Nano sensors derived from organic scaffolds present a distinctive and environmentally friendly approach for the detection of mercury ions, utilizing the versatility and tunability of organic materials. These sensors are crafted from organic scaffolds, often derived from polymers or biomolecules, which are functionalized to selectively interact with mercury ions. The organic nature of these scaffolds ensures biocompatibility and allows for tailored designs, imparting selectivity, and sensitivity to the sensing platform. Organic nano sensors exhibit a unique advantage in terms of ease of modification and integration into various detection mechanisms. For instance, a research investigation utilized rhodamine-derived organic compound (RHD) to construct an RHD-NPs platform tailored for detecting Hg(ii) within water samples. This platform exhibited distinct absorption bands at 325 nm and 269 nm, recognized to n–π* and π–π* transitions, correspondingly. Upon introducing Hg(ii) to the RHD-NPs solution, there was an increase in absorbance at 269 nm, coupled with the a new absorption band appears at 562 nm, attributable to the ring-opening mechanism inherent in the rhodamine structure. Notably, the RHD-NPs displayed a fine linear response, exhibits a LOD of 0.0165 nM (1.6 × 10^−7^ M). This sensor platform proved effective in real water samples for Hg(ii) detection. Furthermore, the study devised a smartphone-based method and a visual colorimetric strip on paper for the convenient sensing of Hg(ii) (Scheme 22).^142^

Moreover, Hg(ii) was detected using fluorescent organic nanoparticles (FONs) possessing dual-emission and two-photon absorption properties. Amphiphilic furyl diketopyrrolopyrrole (DPP1) and tetraphenylethylene co-assembled in water to form these FONs. In water-soluble FONs, DPP1 functioned as an efficient Hg(ii) receptor and a material for two-photon absorption, generating light at 540 nm. In the meantime, the nanostructure was stabilized by TPE1, a common chemical for aggregation-induced emission (AIE), and an identifiable fluorescent signal was generated at 470 nm, which functioned as the internal reference point. These hybrid FONs were successfully used to detect Hg(ii) in water by ratiometric and two-photon fluorescence methods. They demonstrated good selectivity and sensitivity, with a detection limit of 13 nM. Moreover, these FONs were utilized for cell imaging, indicating their promising potential in biological sensing applications.^143^ Fluorescent 2-(thiophen-2-yl)-2,3-dihydroquinazolin-4(1*H*)-one (TDHQ) derivative-based organic nanoparticles (TDHQNPs) were recently developed for mercury sensing by Saubai B. Wakshe *et al.*^144^ Under ideal conditions, fluorescence studies showed a specific and sensitive response only to Hg(ii) among the tested coexisting species, as confirmed by fluorescence titration experiments. Due to electrostatic metal–ligand interactions, the interaction between TDHQNPs and Hg(ii) led TDHQNPs to fluoresce less, resulting in a LOD of 0.00329 μg mL^−1^ within the linear concentration range of 0 to 100 μg mL^−1^. A comparative overview of organic scaffold derived nanosensor presented in Table 10.

**Table 10:** An overview organic scaffold derived nano-sensors for sensing of mercury metal ion

Entry	Nano sensor	Sensing approach	Linear range	LOD	Ref.
1	RHD-NPs	Ring-opening in Rh	—	6.56 nM	142
2	FONs	Ratiometric and two-photon fluorescence	—	13 nM	143
3	TDHQNPs	Fluorescence quenching	0 to 100 μg. mL^−1^	0.00329 μg. mL^−1^	144

### 2.10. Miscellaneous nano-sensor for mercury sensing

In addition to the discussed categories, researchers have also been developed some captivating nanosensors for mercury sensing in the past few years. For instance, unique fluorescent polydopamine nanoparticles (PDANPs) were fabricated for the successive detection of Hg(ii) ions and l-ascorbic acid (AA). The nanosenor utilizes coordination effects and redox reactions. The PDANPs exhibited a fluorescent quenching response to Hg(ii) ions *via* a coordination effect. Upon simultaneous addition of ascorbic acid (AA) and Hg(ii) to the solution, Hg(ii) underwent rapid reduction to elemental mercury (Hg^0^), facilitated by AA acting as a reductant. This process resulted in the oxidation of AA to dehydroascorbic acid (DHAA). As a result, the fluorescence intensity of the PDANPs gradually increased with increasing concentrations of AA. This process is attributable to the reduction of the quenching effect. The fluorescent PDA-NP-based nanosensor demonstrated outstanding sensitivity and selectivity towards Hg(ii) and AA tests.^145^ Furthermore, a portable nanosensor has been devised for Hg(ii) sensing with high sensitivity. Utilizing CuS nanozyme as the Hg(ii) recognition component. The integration of enrichment and detection techniques in this design is customizable, allowing the sensor to cover a broad sensing range from 50 ppt to 400 ppb while maintaining great sensitivity and a 50 ppt minimum detectable Hg(ii) concentration. This developed nanosensor demonstrates the capability to analyze Hg(ii) levels in real samples (food and environmental) with adequate accuracy and reproducibility, exhibiting a deviation of less than 10% and achieving an approximate 82% recovery rate.^146^

A fluorescent sensor have designed to detect both trithiocyanuric acid (TCA) and Hg(ii) through competitive interactions, showcasing its versatility and efficacy in simultaneous detection applications. The interactions were against coordinative contacts between TCA and Hg(ii) ions, involving non-covalent stacking interactions between g-C_3_N_4_ and TCA. When TCA was added to a g-C_3_N_4_ nanosheet solution without Hg(ii) ions, it interacted with the nanosheets through π–π and H-bonding interactions, which led to the fluorescence being quenched. Nevertheless, the introduction of Hg(ii) ions led to the re-establishment of fluorescence in the TCA–g-C_3_N_4_ nanosheet hybrid system. This occurred through the coordination of Hg(ii) with TCA *via* the sulphur (S) atoms, consequently interrupting the stacking contact between TCA and g-C_3_N_4_. The detection method exhibited notable selectivity in identifying TCA and Hg(ii) ions accurately.^147^ Furthermore, a publication describes the development of a low-cost paper-based aptasensor capable of monitoring both Hg(ii) and Ag(i). The sensing array's method is based on the conformational changes of Hg(ii) and Ag(i)-specific aptamers, which are released from the graphene oxide (GO) surface after the target material is injected onto the sensing platform. By tracking variations in fluorescence recovery against ion concentrations, it was possible to detect Hg(ii) and Ag(i) concentrations as low as 1.33 and 1.01 pM, respectively. Within around 10 minutes, the paper-based aptasensor detects all the ions simultaneously. The aptasensor is successfully used to detect Hg(ii) and Ag(i) levels in milk, human serum, and water.^148^

A new hybrid nanomaterial, graphene oxide-lanthanide fluoride (NaxLiyGdF4:Tb^3+^@PMA@Phen@GO), has been investigated for Hg(ii) detection. The hybrid nanomaterial is fabricated *via* functionalizing the as-prepared NaxLiyGdF4:Tb^3+^ with pyromellitic acid (PMA) and 1,10-phenanthroline (Phen), followed by depositing it on the surface of graphene oxide (GO) using a non-covalent method. Due to its exceptional optical characteristics, sensor is employed to detect toxic metal ions such as Hg, Co, Ni, Mn, and Pb, among others. Luminescent experiments reveal that the luminescent nanosensor has a preferential “turn-off” response to Hg(ii) over other hazardous metals. Under optimal conditions, the sensor can detect Hg(ii) ions with an LOD of 1.03 ppm. The developed nanocomposite demonstrates an enhanced interaction with Hg(ii) attributed to the phen ligand. This interaction disrupts the antenna effect that arises from the phen ligand.^149^ Similarly, a dual-mode nanosensor has been established to enable both SERS and fluorescence detection, specifically for detecting Hg(ii) ions. Human telomeric G-quadruplex DNA's conformational switching is important to the signal transduction pathway, serving as a pivotal step in the detection process. Through utilization of T–Hg(ii)–T coordination chemistry, the sensor demonstrates effectiveness in Hg(ii) ions detection in both buffer solution and real samples. It attains an outstanding LOD of 1 ppt, outperforming conventional optical sensors by a magnitude of 100 in terms of sensitivity.^150^

**Fig. 16:**
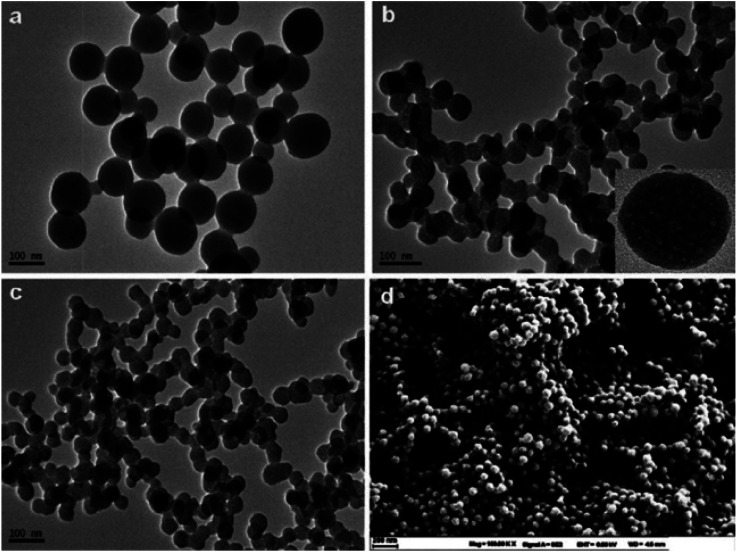
TEM images of (a) Ad/Tb, (b), Ad/Tb/DPA, (c) Ad/Tb/DPA in the presence of Hg(ii) ions (d) SEM image of Ad/Tb/DPA (reproduced from ref. 153 with permission from American Chemical Society, Copyright © 2012).

**Scheme 23:**
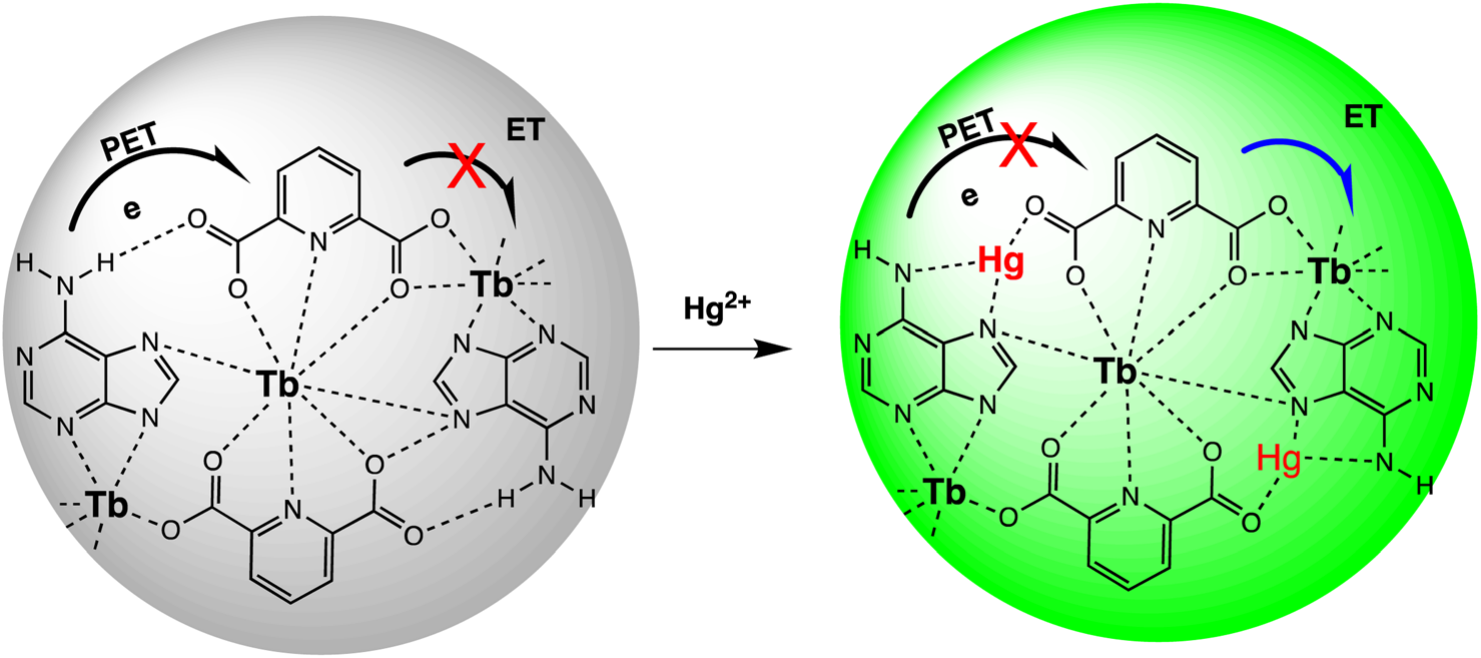
Graphic representation of Hg(ii) sensing using Ad/Tb/DPA *via* PET.

Further, in a report, well-ordered silica-based SBA-15 NPs were functionalized with 3-isocyanatopropyltriethoxysilane and dansyl cadaverine to develop a hybrid material for Hg(ii) ions detection. The solid-state nanosensor exhibits a highly organized 2-D mesoporous structure with a pore size of 8.7 nm. It possesses a distinct morphology comprising nanoscale slice-like bundles, along with covalent interactions between the dansyl fluorophore and the silica surface. These hybrid materials demonstrate high sensitivity and selectivity, serving as fluorescent chemosensors for the detection of Hg(ii).^151^ Similarly, molybdenum disulfide (MoS_2_) nanosheets were constructed and utilized for the simultaneous detection of Hg(ii) and Ag(i) ions in an aqueous solution. The sensing mechanism of the sensor involves the generation of T–Hg(ii)–T or C–Ag(i)–C mismatches through single-stranded DNA (ssDNA) that is abundant in thymine (T) or cytosine (C). This process leads to the formation of stable double-stranded DNA (dsDNA) structures. The sensor has excessive sensitivity and robust selectivity for Hg(ii) and Ag(i), with LOD of 6.8 nM and 8.9 nM, correspondingly.^152^ Based on inhibiting the intramolecular PET process from adenine to DPA, a sensor named Ad/Tb CPNPs was developed for Hg(ii) sensing. The morphology of these lanthanide CPNPs was analyzed *via* TEM and SEM analysis, as shown in Fig. 16, revealing their spherical shape. Furthermore, the binding sites of sensor for Hg(ii) ions were investigated by comparing the FTIR spectra of sensor in the absence and presence of Hg(ii) ions. Furthermore, a shift has been noted in the CN stretching vibration and NH_2_ scissoring vibration of adenine within the sensor, transitioning from 1075 and 1396 cm^−1^ to 1090 and 1384 cm^−1^, respectively. Similarly, DPA's carboxyl asymmetric vibration changed from 1699 cm^−1^ to 1623 cm^−1^, indicating that it interacted with the Hg(ii) ions through its carboxylic acid group. Further confirmation was provided regarding the inhibition of the intramolecular PET from the adenine nitrogen to DPA due to the formation of the Hg–Ad/Tb/DPA complex, resulting in fluorescence enhancement, as illustrated in Scheme 23. Ad, Tb, and DPA CPNPs demonstrated remarkable selectivity and sensitivity. Achieving a LOD as low as 0.2 nM. Additionally, sensor exhibited a sufficiently long fluorescence lifetime suitable for time-resolved fluorescence assays due to the incorporation of Tb^3+^ ions. This characteristic is particularly beneficial for detecting bio-samples with autofluorescence.^153^

**Scheme 24:**
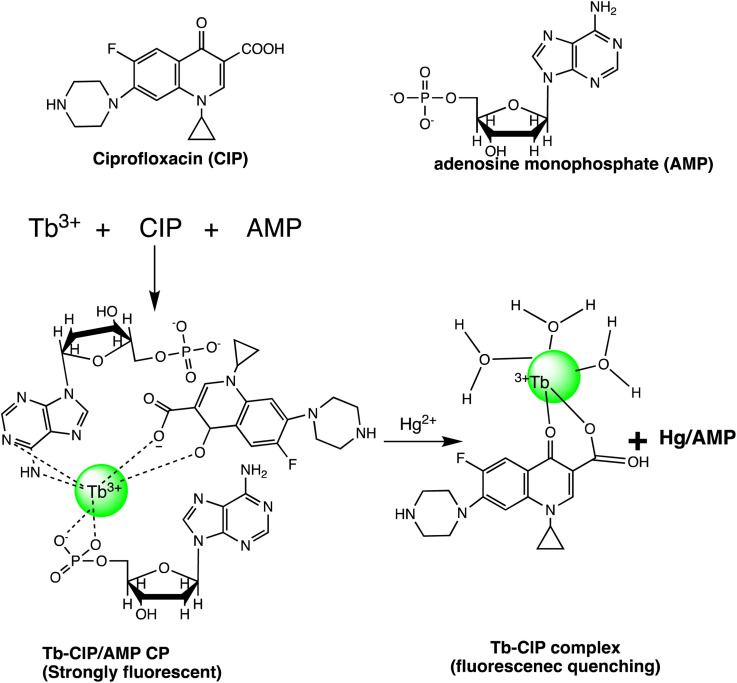
Schematic representation of Tb–CIP/AMP CP preparation and Hg(ii) sensing.

Zeng *et al.* conducted research on a FRET-based sensor designed for detecting Hg(ii) ions. Within this sensor setup, fluorescein isothiocyanate operates as the donor, while a spirolactam rhodamine derivative acts as the probe for detecting mercury ions. Both scaffolds were covalently linked to polyethyleneimine and polyacrylic acid, respectively. The formation of the ratiometric sensing system involved layering polyelectrolytes containing donors and probes onto negatively charged polymer particles through a layer-by-layer approach. Under optimized conditions, sensor exhibit a stable response to Hg(ii) ions in aqueous media with a LOD of 200 nM, and applicable over pH range of 4.6–7.3.^154^ In a recent report, Tb–CIP/AMP was developed for Hg(ii) detection using lanthanide ions as the metal nodes, CIP as the ligand molecule and bridging linker, and AMP as the recognizer. The sensor emits a strong green luminescence due to the incorporation of AMP, which removes the coordinated H_2_O molecules and protects against the damping effect of the OH vibration in the Tb^3+^–H_2_O molecules. Due to the particular coordination interaction between AMP and Hg(ii) ions, the introduction of Hg(ii) ions results in a significant fluorescence quenching of the sensor. The probe proven remarkable selectivity and sensitivity for Hg(ii) ions, achieving a LOD of 0.16 nM. Moreover, the probe exhibits a prolonged fluorescence lifetime of milliseconds. It has proven to be an efficient method for detecting mercury(ii) in human urine samples and drinking water, yielding satisfactory outcomes compared to conventional methods (Scheme 24). A overview of miscellaneous nano-sensors is presented in Table 11.^155^

**Table 11:** An overview of miscellaneous nano-sensors for sensing of mercury metal ion

Entry	Nano sensor	Sensing approach	LOD	Ref.
1	CuS HNSs	Colorimetric	50 ppt	146
2	Paper-based apta-sensor	Fluorescent	1.33 pM	148
3	NaxLiyGdF4:Tb^3+@^PMA@Phen@GO	Fluorescent	1.03 ppm	149
4	DNA-metal nanoparticle conjugates	SERS	1 ppt	150
5	Molybdenum disulphide (MoS_2_) nanosheets	Fluorescent	6.8 nM	152
6	Ad/Tb/DPA CPNPs	Fluorescent	0.2 nM	153
7	Polymer nanoparticle	FRET	200 nM	154
8	Tb–CIP/AMP	Fluorescent	0.16 nM	155

## 3. Future perspective and conclusions

In conclusion, the development of nano-sensors for mercury detection holds great potential in addressing the environmental and health risks associated with mercury contamination. These sensors offer exceptional sensitivity, selectivity, and quick reaction times, making them ideal for monitoring mercury levels in various environmental samples. The integration of nanotechnology in mercury detection has the potential to revolutionize environmental monitoring and safeguard public health. Future research in this field should focus on improving the detection capabilities of nano-sensors by enhancing their selectivity and sensitivity through functionalization processes and the development of hybrid nanomaterials. Additionally, efforts should be made to make the sensors more sustainable and economically efficient to ensure their widespread implementation in different environments. The integration of nano-sensors with artificial intelligence (AI) and IoT technologies will further enhance their usability by enabling real-time monitoring and data analysis. This will facilitate prompt decision-making and action to mitigate the risks posed by mercury contamination.

However, there are still challenges that need to be addressed. The cost, specificity, and production of nanomaterials, as well as the ability to detect multiple analytes, are limitations that require careful consideration. Overall, the development of nano-sensors for mercury detection represents a significant advancement in environmental monitoring and public health protection. By promoting innovation and multidisciplinary collaboration, the scientific community can continue to improve these technologies and effectively address the issues posed by mercury pollution. The ultimate goal is to create advanced technologies that efficiently detect and mitigate the risks associated with mercury contamination, thereby protecting the environment and enhancing public health.

## Data availability

No primary research results, software or code have been included, and no new data were generated or analyzed as part of this review. All information cited within this review is derived from existing literature and publicly available sources.

## Conflicts of interest

There are no conflicts to declare.

## Supplementary Material
